# From Global Emissions to Local Impacts: Spatially Explicit Modeling of Ocean Acidification in Life Cycle Assessment

**DOI:** 10.1021/acs.est.5c02069

**Published:** 2025-10-16

**Authors:** Sedona R. Anderson, Konstantin Stadler, Francesca Verones

**Affiliations:** Industrial Ecology Programme, Department of Energy and Process Engineering, 8018 NTNU, 7491 Trondheim, Norway

**Keywords:** ocean acidification, LCA, characterization factor, LCIA, impact model, ecosystem damage, marine, biodiversity

## Abstract

Ocean acidification poses a critical threat to marine ecosystems. While life cycle assessment frameworks provide a method for assessing and combatting many anthropogenic impacts, marine impact models remain underdeveloped compared to their terrestrial counterparts. This study presents the first spatially explicit characterization model for quantifying the impacts of ocean acidification that includes both midpoint and endpoint characterization factors (CFs). Midpoint CFs were spatially delineated by using marine ecoregions and Food and Agriculture Organization fishing areas, leveraging spatially explicit fate and fate sensitivity factors. Endpoint CFs were calculated using species sensitivity distributions that include species across a range of calcification levels, climate zones, and trophic levels. Results demonstrate significant geographic variability in ocean acidification impacts, with polar regions showing heightened vulnerability. Our findings emphasize the need for spatially explicit modeling to account for the diverse biogeochemical and ecological responses to ocean acidification. This work advances marine impact assessment by integrating spatial and biological complexity, providing critical tools for quantifying ocean acidification’s global ecological and economic consequences.

## Graphical abstract

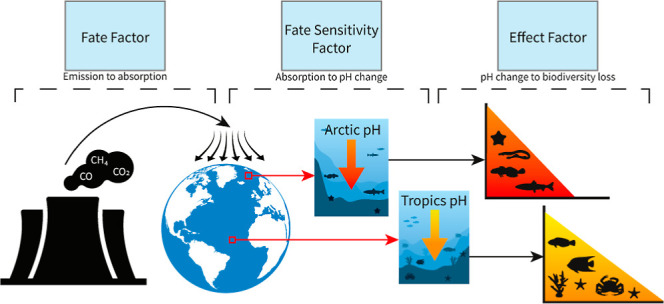

## 1 Introduction

The ocean is the world’s largest carbon sink, absorbing roughly 30% of anthropogenic greenhouse gas (GHG) emissions.^1,2^ When GHGs from the atmosphere meet the ocean surface, they react with water and undergo chemical dissociation that ultimately results in decreased pH and reduced availability of carbonate to biological systems.^3^ This process is known as ocean acidification.

The current rate of ocean acidification is more severe than any seen in at least 55 million years and continues to accelerate with unabated GHG emissions.^4^ Since preindustrial measurements, marine pH has fallen by roughly 0.1 units to the current average pH of 8.1 ^1,5,6^ and is predicted to decrease by 0.3–0.4 units by the end of the century.^1,5^ This could mean up to a 150% increase in surface ocean acidity and the most rapid and severe change marine ecosystems will have experienced in tens of millions of years.^3,4^

Ocean acidification affects the survival of marine organisms in a variety of ways. Strongly calcifying species are affected most drastically through the weakening of shells or exoskeletons.^4,7^ A growing body of research suggests that noncalcifying species, such as fish, may also be affected by impacts, including tissue acidosis and behavioral change.^7,8^ Both calcifying and noncalcifying organisms are affected via reef collapse, decreased larval survival rates, and food web instabilities.^4,7^

In addition to pressure from rising GHG emissions, there has also been a significant increase in economic activity in the ocean.^9^ While encouraging marine economic expansion, the European Green Deal and the EU Blue Economy Policy, among other initiatives,^9–11^ simultaneously recognize the importance of preserving the ocean’s ecological integrity in the face of growing environmental pressure. To realize these dual goals, there need to be tools ready that can assess potential environmental impacts on marine ecosystems. Life cycle assessment (LCA) is one such tool for achieving this through the quantification of human impacts on the environment and identification of leverage points for reducing environmental harm.^12^ However, within LCA, there are significantly fewer marine impact models compared to their terrestrial counterparts.^13^ In order to achieve the joint objectives of marine economic expansion and biological conservation, marine impact models must be expanded in scope and detail.

The best available models for ocean acidification impacts include a spatially generic midpoint model for CO, CO_2_, and CH_4_ emissions^2^ and an endpoint model calculated using the aforementioned spatially generic midpoint combined with an effect model that is spatially delineated for three regions and has separate effect factors for slightly and strongly calcifying species.^14^ The major limitation of current models is that there has been no midpoint (and thus no endpoint) that is fully spatially defined. The existing midpoint model treats the entire marine realm as homogeneous, which presents a critical knowledge gap, as spatially generic models are not sufficient to accurately predict impacts from ocean acidification. Increased absorption of GHGs affects marine ecosystems differently across the globe due to complex biogeochemical cycles, including factors such as carbonate chemistry variability, biological activity (photosynthesis and respiration), temperature and salinity, nutrient availability, geographic and seasonal variations, as well as human impacts and ecosystem disruption.^4^ Due to these factors, GHGs absorbed by the ocean will result in different changes in pH and carbonate availability in different geographical locations with different effects on species and ecosystems.^15,16^ The existing effect model produces abnormally large effect factors, as acknowledged by the authors, and uses a bucketing approach for creating species sensitivity distribution (SSD) curves that falls outside the standard method.^14^ Additionally, research on how ocean acidification impacts slightly calcifying and non-calcifying organisms is rapidly advancing, and SSDs and their resulting effect factors should be frequently updated to reflect this.

In this study, we build upon existing models to improve their species coverage, spatial delineation, and accuracy to finally arrive at a complete and operational spatially explicit characterization model for ocean acidification for both midpoint and endpoint.

## 2 Methodology

The life cycle impact assessment (LCIA) phase of an LCA is when the lifetime environmental impacts of a product or service are quantified and interpreted.^17^ This is done using characterization factors (CFs), derived from impact models, to convert an elementary flow into various category indicators. Models can be built to the midpoint or endpoint level. The endpoint method aggregates impacts to an “Area of Protection” (ecosystem damage, in our study).^17^ The midpoint method aggregates impacts to a specified level of cause-effect between the emission and the endpoint level.^17^ Our model for ocean acidification contains both midpoint and endpoint levels and is quantified in three “steps” from emission to impact (Figure 1). The fate factor (FF) quantifies the pathway from emission of the GHG to its contact with marine water and subsequent spCO_2_ change, the fate sensitivity factor (FSF) quantifies the impact of spCO_2_ change on pH, and the effect factor quantifies the impact of pH change on marine species.

**Figure 1:**
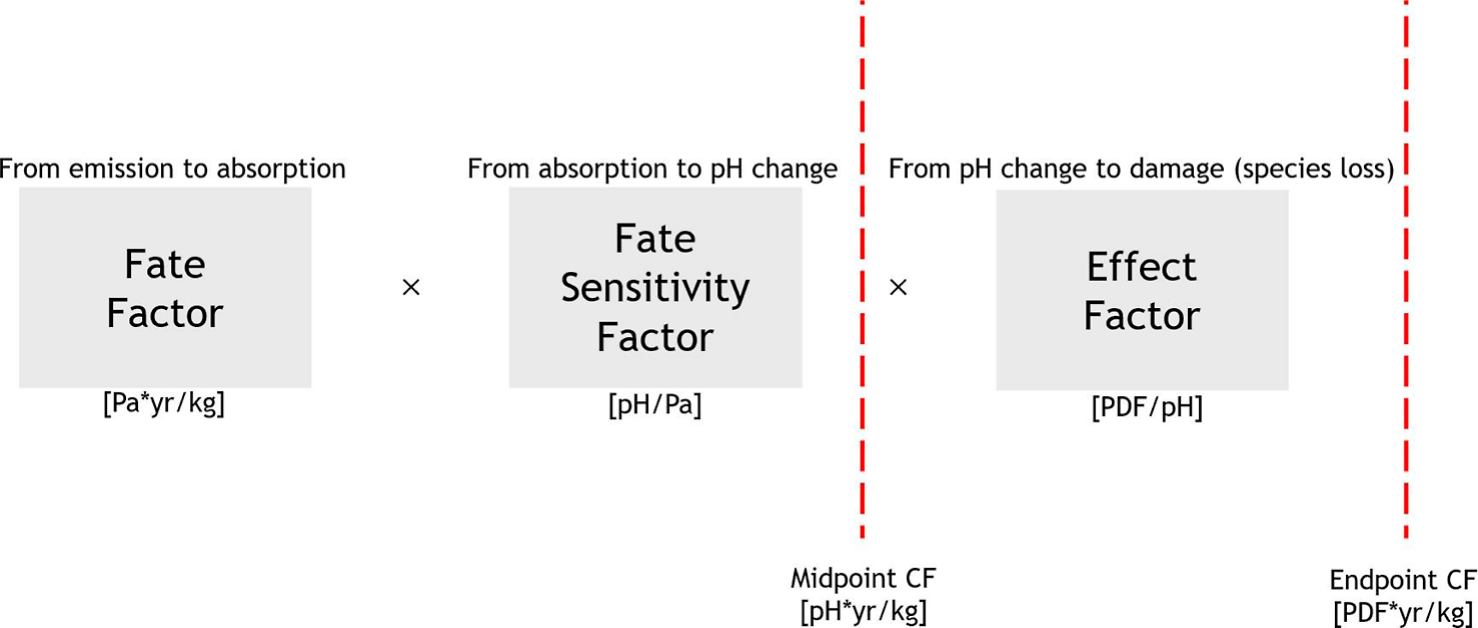
Model framework for midpoint and endpoint CFs. Below each modeling step, the unit of the resulting factor is indicated in square brackets. Potentially disappeared fraction (PDF) indicates the “potentially disappeared fraction of species” commonly used in LCIA modeling.

The midpoint CF quantifies the change in marine pH given an emission, and the endpoint quantifies ecosystem damage through the metric PDF of species given an emission.

Python version 3.13.0, ArcGIS Pro version 3.3.0,^18^ and R version 4.4.1 ^19^ were used for data processing. Packages and their versions used in Python and in R can be found in Supporting Information (Section 1 and Table S1).

### 2.1 Midpoint CF Model

The model for midpoint CFs is made up of two components, the FF and the FSF (eq 1)­

1 $${CF}_{midpoint,j,i}={FF}_{j,i}\times \left(-{FSF}_{j}\right)$$

where the midpoint CF [pH·yr/kg] for area *j* and substance *i* is the product of the FF [Pa·yr/kg] for area *j* and substance *i* and the FSF [pH/Pa] for area *j*. In our case, the FSFs are negative (as the pH declines); thus, an additional negative is used in the midpoint equation, as the CFs must be positive to show that there is a net impact.

Midpoint CFs have been calculated for the substances carbon dioxide (CO_2_), carbon monoxide (CO), and methane (CH_4_). They were spatially delineated for 232 marine ecoregions^20^ for coastal waters and 18 Food and Agriculture Organization (FAO) major fishing areas^21^ for open ocean.

Marine ecoregions are biogeographic units of cohesive species composition, shaped predominantly by the same forcing agents included in marine biogeochemical cycles (i.e., upwelling, temperature, nutrient cycles, and currents),^20^ and thus make a natural spatial unit for delineation. However, they are limited to coastal environments. Lacking a similarly comprehensive biogeographic unit for the open ocean, the FAO major fishing areas were chosen. FAO major fishing areas are defined using inputs from international fisheries on both geopolitical and ecological boundaries.^22^ The area overlapping with marine ecoregions was removed from FAO major fishing areas using ArcGIS to avoid double counting.

#### 2.1.1 Fate Factor

The FF quantifies the pathway from the emission of GHGs to absorption by marine waters. The formula for calculating the Revelle factor (RF), which quantifies the buffer capacity of the marine carbonate system,^6^ can be solved for calculating the change in surface partial pressure CO_2_ (ΔspCO_2_), given a known RF (eq 2):

2 $$\Delta {spCO}_{2}={spCO}_{2orig}\times RF\times \left(\frac{\Delta DIC}{{DIC}_{orig}}\right)$$

Global surface ocean RF values were sourced from the National Oceanic and Atmospheric Administration Ocean Carbon and Acidification Data System.^6^ Monthly values spanning the entirety of 2020 were used to account for seasonal fluctuations, at a 1 arc degree resolution. The RF data were generated by linking present-day in situ observations with an Earth System Model and include modeled values for Representative Concentration Pathways (RCPs) 2.6, 4.5, 6.0, and 8.5.^6^ We chose to use values modeled under RCP 4.5, as it represents a stabilization pathway that is broadly consistent with current emissions trends under moderate mitigation.^6,23^ Values were arithmetically averaged per spatial unit.

To evaluate the influence of this choice on the results, we compared the RF and corresponding midpoint CFs between the RCP 4.5 and the RCP 6.0. The average change in RF from RCP6.0 to RCP4.5 was −0.0027 [-], with an associated average change in CF of −2.49 × 10^–17^. This yields an average sensitivity of 2.92 × 10^–15^ impact units per unit change in the RF. In practical terms, even a full unit decrease in RF would result in a negligible change in the midpoint CF. These results support the conclusion that the CFs are effectively insensitive to small variations in RF across plausible midrange RCPs and justify the use of a single representative scenario in this study.

The variables DIC_orig_ (dissolved inorganic carbon (DIC)) [mol/m^3^] and spCO_2orig_ (surface partial pressure CO_2_) [Pa] are sourced from the Global Ocean Biogeochemistry Analysis and Forecast hosted on Copernicus,^24^ which is powered by the marine biogeochemical model NEMO-PISCES v3.6.^25^ Monthly global values from November 2021 to December 2022 were downloaded with a resolution of 0.25 arc degrees. This date range was selected as it was the closest available match to the RF data. Though the resolution between these data and the RF data is different, values were again arithmetically averaged per spatial unit, and as such, all values used in the equation were of the same regional resolution.

The value for ΔDIC in eq 2, relative to DIC_orig_, can be calculated with eq 3, given an emission of substance *i* (eq 3):

3 $$\Delta DIC=\frac{\left(\frac{{\Delta E}_{i}\times {DF}_{i}}{M_{{CO}_{2}}}\right)}{A}$$

The dissolution factor (DF_*i*_) [−] in eq 3 quantifies how much of the emission of substance *i* (*ΔE*_*i*_) [kg/yr] will eventually dissolve in the ocean as CO_2_. The dissolution factors for CO_2_, CO, and CH_4_, respectively, are 0.27, 0.24, and 0.23. 27.5% of CO_2_ emitted to the atmosphere dissolves in the ocean, where 0.225% is buried in the sediment. CO and CH_4_ both convert to CO_2_ in the troposphere before contributing to ocean acidification as CO_2_ dissolves with seawater. 87.1% of carbon monoxide reaches the troposphere and converts to carbon dioxide. 87.8% of methane reaches the troposphere, of which 95% reacts to carbon monoxide and then to carbon dioxide. Once the gases have been converted to carbon dioxide in the atmosphere, the CO_2_ dissolution rates apply. These values are calculated from the spatially generic fate model pathway by Bach et al.^2^ As CO and CH_4_ convert to CO_2_ in the troposphere, only 1 M mass constant [kg/mol] is used, that of CO_2_, given as 4.4 × 10^–2^ kg/mol.^26^ The dissolution factors account for the conversion of CH_4_ to CO_2_. It is assumed that an emission will be well-mixed and may be absorbed by the ocean at any location, thus *A* represents the surface volume of the open ocean free of ice shelves, equaling 361 × 10^6^ m^3^.^27^

#### 2.1.2 Fate Sensitivity Factor

The FSF reflects the change in the considered environmental compartment when an emitted substance enters it. This was calculated using linear regression modeling of the relationship of pH with respect to spCO_2_ and repeated for each spatial unit individually. pH and spCO_2_ values were sourced from the Global Ocean Biogeochemistry Analysis and Forecast hosted on Copernicus, powered by NEMO v3.6.^24^ Monthly global values from November 2021 to December 2022 were downloaded with a resolution of 0.25 arc degrees.

#### 2.1.3 Characterization Factors

Lastly, midpoint CFs [pH·yr/kg] were calculated by multiplying the fate [Pa·yr/kg] and FSFs [pH/Pa] for each emission substance (eq 1). Calculations resulted in 750 CFs (232 ecoregions and 18 FAO fishing areas for each of the three considered GHGs). The area of each spatial unit was quantified in km^2^ using geodesic calculations on the ellipsoid in EPSG:4326 and used to weight globalized midpoint CFs. The aggregated CFs (CF_agg,i_) were then normalized with respect to the reference substance, CO_2_, to achieve final, global values in CO_2_-equivalents (CF_norm,i_):

4 $${CF}_{norm,i}=\frac{{CF}_{agg,i}}{{CF}_{agg,{CO}_{2}}}$$

### 2.2 Effect Factor Model

Combining the spatially explicit midpoint CFs with spatialized effect factors produces endpoint CFs at the level of the FAO regions or marine ecoregions. Effect factors convert a midpoint impact to an endpoint damage, which, within our study, is quantified in the PDF of the species. The SSD is a statistical method used to estimate what pH is hazardous to *x* % of all species (HC*x*).

In order to build SSD curves, experimental data on the toxicity responses of various species are required. Within our study, the methodology presented by Azevedo et al.^48^ was used to derive the pH at which a 50% reduction of a vital trait (such as growth or reproduction) occurs (pH_50_), as well as the pH at which a 10% reduction of a vital trait occurs (pH_10_) for each experiment. Logistic regression curve fitting was used to derive the pH values where 50% and 10% of a species were affected (pH_50_ and pH_10_ values, respectively).

Once HC*x* is derived from the SSD curves, it can be used to calculate the effect factors (eqs 8 and 9).

#### 2.2.1 Data Collection

Our aim was to collect species response data at various levels of ocean acidification across a broad spectrum of regions and calcification levels. We utilized data compilation from the Ocean Acidification International Coordination Centre, containing studies of ocean acidification effects on organisms, published from 2008 to 2023, to find relevant experiments. The data set is hosted on PANGAEA.^28^ Experiments involving kingdoms other than animalia and testing multiple stressors (e.g., combining acidification with elevated temperatures or increased metal toxicities) were excluded. To use Azevedo et al.’s^48^ method, experiments had to have at least three pH test conditions, as the logistic function (eq 6) has two parameters and requires one degree of freedom. This search resulted in 24 usable studies with data points for 32 species in total (study references can be found in Supporting Information, Section 2).

To ensure representation of a variety of species, studies were classified by calcification level, region, and trophic level (Table S2). For the calcification level, we have defined strongly calcifying species as species that have major external calcifying elements (i.e., carbonate exoskeleton or shell), slightly calcifying species as having major internal calcifying elements (i.e., carbonate cuttlebone or endoskeleton), and noncalcifying species as having minor internal calcifying elements (i.e., otoliths or calcareous spicules). Although nearly every species has some kind of calcifying biological function, we have decided to call the lowest calcification category “non-calcifying” in line with existing literature.^29^

For the effect factor calculation, we delineated 4 climate zones: polar, temperate, subtropical, and tropical.^30^ Species may belong to more than one climate zone. Three trophic levels were distinguished: primary consumer (level 2), secondary consumer (level 3), and tertiary consumer (level 4). Trophic level 1 contains plankton and algae that are affected by ocean acidification; however, we were unable to find any studies without the dual stressors of pH and light exposure, and thus, these experiments were excluded. Trophic level 5, comprised of apex predators such as sharks or whales, was excluded as no experiments have yet shown that ocean acidification affects them directly. The exclusion of trophic levels 1 and 5 represents a limitation of the current study and should be considered when comparing across endpoint impacts, where the set of species included may differ.

#### 2.2.2 Derivation of pH_50_ and pH_10_ Values

To calculate pH_50_ and pH_10_ values for each experiment, we first calculate the empirical relative response, a normalized response rate

5a $${eRR}_{t}=1-\frac{R_{c}}{R_{t}}$$

where *R*_c_ is the reference response at pH_c_ (the control condition) and *R*_t_ is the relative response at pH_*t*_ (the test condition). Equation 5a represents a scenario where the reduction of a vital trait is represented by an increasing response, for example, the bioerosion rate. If the experiment reported a decreasing response (e.g., reduced calcification, fertilization, and degustation), the equation was modified:

5b $${eRR}_{t}=1-\frac{R_{t}}{R_{c}}$$

where the relative response at pH_*t*_ (*R*_t_) is now the numerator and the reference response at pH_c_ (*R*_c_) is now the denominator.

Next, eRR_t_ and corresponding pH_*t*_ values were used to fit a logistic regression with the output variables pH_50_ and β

6 $${cRR}_{t}=\frac{1}{1+10^{\left({pH}_{50}-{pH}_{t}/\beta \right)}}$$

where cRR_t_ is the calculated relative response for pH_*t*_, pH_50_ is the pH at which a 50% reduction occurs in a vital function of the studied species, and β is the slope of the logistic regression. In line with the precautionary principle, it is now recommended to use the effective concentration where a 10% reduction occurs in a vital function;^31^ thus, the pH_10_ value was also calculated using the previously derived pH_50_ and β values. In eq 7, the constant 9 is included as a scaling factor, following Azevedo et al.:^48^

7 $${pH}_{10}=\beta \times \log_{10}9+{pH}_{50}$$

#### 2.2.3 Species Sensitivity Distributions

SSD curves are commonly used to estimate the sensitivity of various species to an environmental stressor, such as ocean acidification, by representing the cumulative proportion of species affected at different concentrations. The SSD curve is then used to determine the hazardous concentration (HC), such as HC20 or HC50, which indicates the pH levels at which 20% or 50% of the species are affected, respectively. In LCIA, it is common to use HC50 values when calculating effect factors.^31^ However, there is growing support for using a lower threshold, such as HC20, as HC50 levels are often well beyond environmentally relevant concentrations.^31^ In this study, we have created SSD curves following both the historical precedent of using HC50 values and a newer approach recommended by Owsianiak et al.^31^ using the value at HC20_pH_10__. This approach uses pH_10_ values (the pH at which there is a 10% reduction in the studied vital function of a species) to build the SSD curve and takes the HC20 value, at which 20% of species are affected at their pH_10_ level.

To construct the SSD curves, pH_50_ or pH_10_ values were used to calculate cumulative proportions, and a logistic regression model with a quasibinomial logit link was used to fit the curve to the pH concentration data. The SSD curves contain data points for 32 different species, encompassing 3 different trophic levels. Two global SSD curves have been constructed, one for HC50_pH_50__ and one for HC20_pH10_, as well as three SSD curves for strongly, slightly, and noncalcifying organisms, and four SSD curves for tropical, subtropical, temperate, and polar regions. The total species representation in each category can be found in the Supporting Information, Table S2.

#### 2.2.4 Effect Factors

There are two ways in which our study varies from a standard ecotoxicology study regarding calculating effect factors. First, whereas a normal ecotoxicology experiment standardly begins with a clearly defined baseline exposure (often assumed to be zero), for ocean acidification, it is not meaningful to consider a “zero exposure” state, since pH is an inherent system property. Instead, we define a reference state corresponding to preindustrial ocean conditions, following the approach taken in other impact categories such as land use. Jiang et al.^6^ provide a model for historical ocean pH. Their data set contains global pre-industrial pH values at a 1 arc degree resolution for every month of the year 1770. We calculated the arithmetical global average of 8.19 to use as the zero-exposure state.

Second, in a standard ecotoxicology study, the higher the exposure value that a species can tolerate, the less sensitive they are. In the case of ocean acidification, the opposite is true: the lower the exposure value (i.e., the more acidic the water) a species can tolerate, the less sensitive they are. We account for this by following the precedent of eutrophication models that work with dissolved oxygen, which face a similar dilemma.^32^ We calculate the sensitivity based on the change (ΔpH) from the reference state to the HC value. Following the approach suggested by Owsianiak et al.,^31^ an effect factor [potentially affected fraction (PAF)/pH] was then calculated using the HC20_pH10_ value, using eq 8:^33^

8 $${EF}_{20}=\frac{0.20}{{{pH}_{orig}-HC20}_{{pH}_{10}}}$$

Following historical precedent, an effect factor was also calculated using the HC50_pH_50__ value, using eq 9:

9 $${EF}_{50}=\frac{0.50}{{{pH}_{orig}-HC50}_{{pH}_{50}}}$$

Effect factors were calculated for each calcification level and region, resulting in 16 effect factors in total. Within the results presented, the global effect factor from eq 8 (EF_20_) was used to calculate endpoint CFs, as it follows the precautionary principle and has the highest representation of species.

The PAF is transformed to the generally used PDF, with an assumption of 1:1 (i.e., species that are affected will also disappear). This is based on the recommendations of the life cycle initiative hosted by the UN Environment, based on work from Oginah.^33^

### 2.3 Endpoint CFs

Endpoint CFs are calculated using eq 10:

10 $${CF}_{endpoint,j,c,i}={FF}_{j,i}\times \left({-FSF}_{j}\right)\times {EF}_{j,c}$$

where the endpoint CF for area *j*, calcification level *c*, and substance *i* is equal to the FF for area *j* and substance *i* (FF_*j*,*i*_) multiplied with the FSF for area *j* (FSF_j_) and the effect factor for area *j* and calcification level *c* (EF_j,c_). The endpoint CF unit is the PDF of species per kilogram emission of substance *i* [PDF·yr/kg]. Calculations resulted in 750 endpoint CFs; 232 for marine ecoregions and 18 for FAO fishing areas, for each of the three studied substances. A globally aggregated endpoint CF was also calculated for each substance using the same method as in the midpoint: the area of each spatial unit was calculated in km^2^ in ArcGIS Pro and used to weight the globalized endpoint CFs.

## 3 Results and Discussion

### 3.1 Midpoint CF Model

Midpoint CFs are the product of two components, the FF and the FSF (eq 1). There are three different sets of FFs, one for each emission substance (CO_2_, CO, and CH_4_). Figure 2B shows the FF values for CO_2_, which range from 5.76 × 10^–12^ Pa/kg (most severe) to 2.50 × 10^–12^ Pa/kg (least severe). The values for CO range from 5.01 × 10^–12^ Pa spCO_2_/kg to 2.18 × 10^–12^ Pa spCO_2_/kg, and those for CH_4_ range from 4.80 × 10^–12^ Pa spCO_2_/kg to 2.09 × 10^–12^ Pa spCO_2_/kg. The value distribution for all emission substances shows more extreme reactions at polar latitudes and less severe reactions closer to the equator, in line with what we would expect based on current literature.^15,16^ All FF values can be found in Tables S3–4 in Supporting Information.

**Figure 2:**
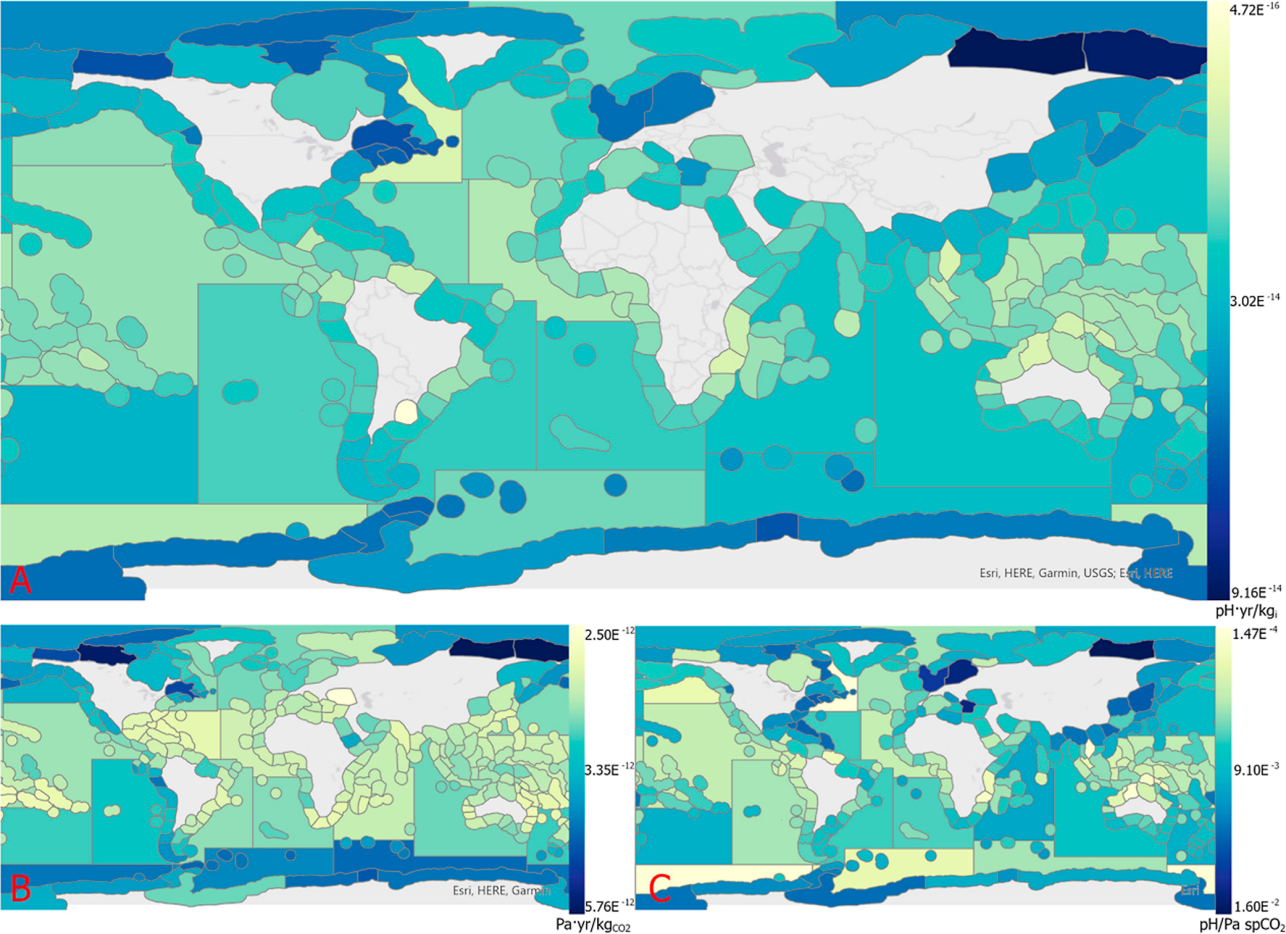
A–C) Figure A is the change in pH per kilogram CO_2_ emitted [pH yr/kg] (midpoint CF_CO_2__) in FAO major fishing zones and marine ecoregions. Figure B is the average change in spCO_2_ [Pa/m^3^] per kg CO_2_ emitted (FF). Figure C is the average change in marine pH per unit increase of spCO_2_ [Pa/m^3^] (FSF). Continent basemap from ESRI (2024).

The second component of midpoint CFs is the FSF. There is only one set of FSFs (Figure 2C) as all considered GHGs convert to CO_2_ in the troposphere.^2^ The FSFs range from 1.60 × 10^–2^ pH/Pa spCO_2_ (most severe) to 1.47 × 10^–4^ pH/Pa spCO_2_ (least severe). These also generally follow expected patterns with some outliers. There are particularly severe FSFs along the North American East Coast and in the Baltic Sea. These align with the locations of the world’s most severe dead zones. The respiration of algal blooms and other organisms, causing hypoxic conditions, also generates excess CO_2_, lowering pH.^34^ This relationship is captured through the pH data and methodology used to calculate the FSFs. The Labrador Sea, off the coast of Greenland, and the Southern Ocean are also outliers. Both regions are subject to unique phenomena that could affect their FSFs. The Labrador Sea has various observed anomalies, including low salinity and oxygen disequilibria that are attributed mainly to cool surface waters from both wind and unique water transport patterns.^35,36^ The Southern Ocean has unique physical mixing due to the Antarctic Circumpolar Current that serves as a link between the Atlantic, Indian, and Pacific Oceans, which could explain the abnormalities in pH response.^37^ The FSF values can be found in Tables S5 and S6 in Supporting Information.

Finally, the midpoint CFs (CF_midpoint_) for CO_2_ are pictured in Figure 2A, ranging from 9.16 × 10^–14^ (most severe) to 4.72 × 10^–16^ (least severe). The CF_midpoint_ for CO ranges from 7.98 × 10^–14^ pH/kg to 4.11 × 10^–16^ pH/kg, and for CH_4_ from 7.64 × 10^–14^ pH/kg to 3.94 × 10^–16^ pH/kg. As with the FF and FSF, they generally follow expected patterns of severity. The global, normalized midpoint CFs, set in relation to the reference substance CO_2_, are 1 kg of CO_2_ Eq/kg_CO2_, 0.87 kg of CO_2_ Eq/kg_CO_, and 0.83 kg of CO_2_ Eq/kg_CH4_. All midpoint CFs can be found in Tables S7 and S8 in Supporting Information.

Fate model values are spatially unique due to biogeochemical cycles made up of complex interactions and feedback mechanisms. System components include temperature, salinity, nutrient availability, and redox, among many others, that all affect acidification trends.^4^ Ocean acidification is most drastic at polar latitudes, specifically northern latitudes, due to the sensitivity of acid–base dissociation at cold temperatures and naturally low carbonate ion concentrations.^15^ Equatorial latitudes have the highest buffering capability for increased pCO_2_, with acidification modulated by the upwelling of lower pCO_2_ waters, as well as high saturation of carbonate materials and biological removal of DIC due to high primary productivity.^16^ Our midpoint results reflect these natural phenomena, with midpoint CFs having higher values toward the poles and lower values nearer to the equator (Figure 2A).

Due to limitations of data availability, there are aspects of the model where accuracy and coverage could be improved in the future, including the difference in ocean acidification potential with respect to depth. The input data for the fate model is composed of surface-level values, with a depth of up to 50 m. This is due to pCO_2_ data only being available at the surface level. However, pH decreases with depth due to the release of CO_2_ from biological processes like respiration, as well as current circulation.^4^ Of the species included in our effect model, four inhabit noncoastal benthic regions: three Echinodermata and one Porifera. As such, impacts for these taxon groups are more likely to be affected by depth-related uncertainty originating from the midpoint model. Advances in depicting effects beyond the surface level could improve the representativeness of calculated impacts.

Depth may also change the suitability of our selected spatial delineations. Through a variability analysis, we found that the FAO fishing area regions, though larger than the MERs, were more homogeneous for the selected data inputs (Supporting Information, Figure S1). This could change if depth were an additional aspect of the model, as the benthic layer of such large regions would likely be more heterogeneous. If depth were included as a variable in a future model, using a smaller regional delineation for the open ocean could increase the accuracy of the model.

Additionally, it is common practice in atmospheric characterization models to assume that GHGs are evenly distributed throughout the atmosphere (“well-mixed”). While the warming effects of GHGs are not bound by the emission location, contact with marine water could be affected by additional spatial and temporal dimensions. If we had an atmospheric distribution model that could be utilized within our FF, it is possible that the ocean surface area of absorption could be smaller depending on the region of emission.

It is important to note that the midpoint CFs presented in our study represent the expected pH change per kilogram of CO_2_ emitted, calculated under specific regional and temporal conditions. If a practitioner would like to update the FFs temporally, they could do so by updating the parameters in eqs 2 and 3 given they have relevant spCO_2_, pH, RF, and DIC data sets. While annual variations are likely to be small, changes over long time spans could be significant.

CFs reflect local, incremental effects in the short term, rather than wholly encompassing the ocean’s complex carbonate buffering system, feedbacks, and gas exchange dynamics over time. The acid–base reactions that form DIC, which is used to calculate the FF, happen on a time scale of tens of seconds,^3^ while atmosphere-ocean equilibrium happens on a scale of several months,^3^ and other processes of the “biological pump” that absorb and rereleases DIC occur over the span of seasons or even years.^38^ The spatial dimension of this model is capable of representing immediate and intermediate impacts but does not incorporate long-term feedbacks, which could change the ocean’s capacity for CO_2_ absorption.^3^ Cumulative global pH change should be interpreted within the broader context of oceanic biogeochemical processes.

### 3.2 Endpoint CF Model

#### 3.2.1 Species Sensitivity Distributions

SSD curves were fitted for two HC levels: HC50_pH_50__ and HC20_pH10_. Both global SSD curves have 32 data points representing 32 different species belonging to three different trophic levels and six phyla, which meets the UNEP-recommended minimum of three species per three trophic levels for SSD derivation.^39^

In Figure 3A, pH_50_ values (the pH at which a 50% reduction occurs in a vital function) are plotted, and the HC50 is derived as 7.47. In Figure 3B, pH_10_ values (the pH at which a 10% reduction occurs in a vital function) are plotted, and the HC20_pH_10__ is derived as 8.04.

**Figure 3:**
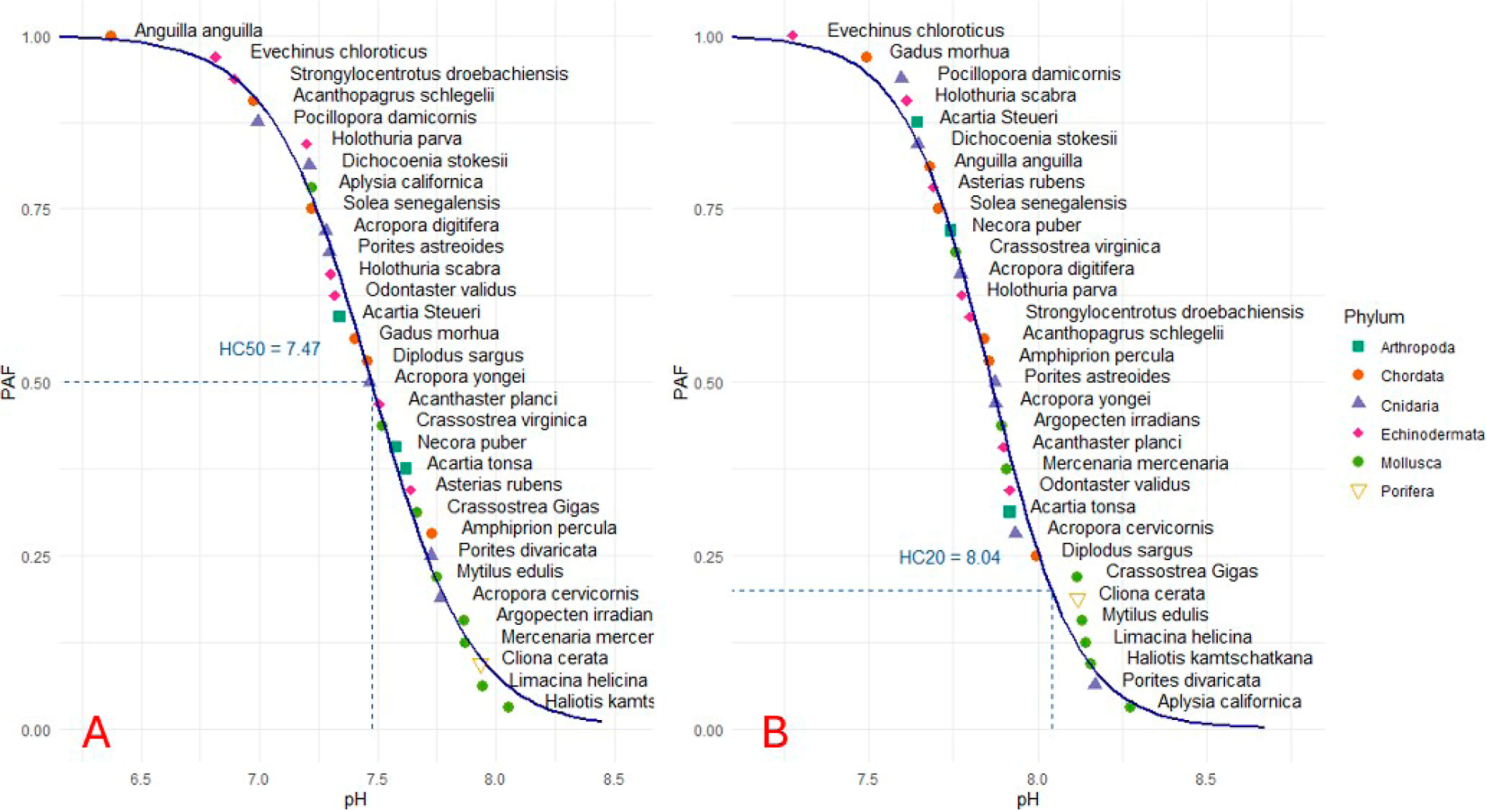
A,B) Figure A pictures the SSD of the pH_50_ values for 32 different marine species. Figure B pictures the SSD of the pH_10_ values for 32 different marine species.

As discussed by Owsianiak et al.,^31^ HC50 values often fall below environmentally relevant thresholds and may not reflect realistic exposure conditions. This is an observation that holds true in our study as well, given that the current average ocean pH is approximately 8.1 and is projected to decline by 0.3–0.4 units by 2100.^1,5^ While we report the HC50 for completeness and consistency with historical practice, we emphasize HC20_pH_10__ as the more environmentally relevant metric. This value (8.04) falls within a realistic future range for ocean pH and is consistent with current recommendations in LCIA.

SSD curves were also fitted for 3 calcification levels: strongly calcifying, slightly calcifying, and noncalcifying (Supporting Information, Figures S2–4), and for 4 regional levels: tropical, subtropical, temperate, and polar (Supporting Information, Figures S5–S8).

#### 3.2.2 Effect Factor

Sixteen effect factors have been calculated; two at the global level, six for the different calcification levels, and eight at the regional level (Table 1). The global EFs contain all 32 species, and the calcification level is irrespective of the region, while the region is irrespective of the calcification level. The global EF_20_ (1.35 PDF/pH) was used in the calculation of endpoint CFs. This represents the most robust EF and follows the current recommendations from the life cycle initiative to use HC20 instead of HC50.^31^

**Table 1:** Effect Factors for Both HC20_pH_10__ and HC50 at the Global Level, as Well as at the Calcification and Regional Levels

		EF20 [PDF/pH]	EF50 [PDF/pH]
	global	1.35 × 10^0^	7.00 × 10^–1^
calcification	strongly	2.46 × 10^0^	8.70 × 10^–1^
	slightly	2.77 × 10^0^	6.18 × 10^–1^
	non	9.82 × 10^–1^	6.63 × 10^–1^
region	tropical	1.05 × 10^0^	6.72 × 10^–1^
	subtropical	1.48 × 10^0^	6.98 × 10^–1^
	temperate	1.84 × 10^0^	7.33 × 10^–1^
	polar	2.61 × 10^0^	7.37 × 10^–1^

The hazardous concentrations of the SSD curves follow expected trends, with strongly calcifying species being more sensitive to ocean acidification and noncalcifying species being less sensitive. The regional SSD curves also generally follow the expected trends, but would benefit from more polar data.

The “slightly” calcifying EF category has the highest uncertainty due to it having the lowest amount of data points. Acknowledging this, we still chose to include this category in order to expand on the traditional binary classification of “noncalcifying” and “calcifying” organisms. Degrees of calcification vary across taxa, and introducing a “slightly calcifying” group allows for more nuanced biological representation. While this category is less certain, it provides a useful starting point for future research and is included here primarily for completeness and transparency.

Additionally, creating a set of SSD curves to compare regions with a high degree of natural pH fluctuation, such as littoral or intertidal areas, with regions having a naturally static pH, could be insightful. Studies have found that animals living in areas with naturally fluctuating pH may adapt better to ocean acidification than those living in regions with little variation.^40–43^ One study found that even among the same species, individuals sourced from a fluctuating environment suffered significantly fewer negative impacts from increased ocean acidification than another population sourced from a more static environment.^41^

It should also be mentioned that 12% of the ocean acidification studies reviewed found that the studied species had a neutral or even favorable response to ocean acidification. In the cases of “favorable” response, this could be due to the species having to exert more energy to maintain homeostasis, resulting in temporarily increased feeding, growth, or breeding activity.^43^ As studies are most commonly acute, with even long-term studies still occurring over one or at most two generations, it is unknown how these temporary increases in energy exertion affect the survival of the species over time.^41^ This demonstrates that although there is scientific consensus that ocean acidification has significant negative effects on strongly calcifying species, we are still learning how it affects slightly or noncalcifying species.^7^

### 3.3 Endpoint CFs

The endpoint CFs for CO_2_ range from 1.23 × 10^–13^ PDF yr/kg (most severe) to 6.36 × 10^–16^ PDF yr/kg (least severe). Endpoint CFs for CO range from 1.07 × 10^–13^ PDF yr/kg to 5.54 × 10^–16^ PDF yr/kg, and for CH_4_ from 1.03 × 10^–13^ PDF yr/kg to 5.30 × 10^–16^ PDF yr/kg. The distribution pattern of endpoint CFs is the same as that of midpoint CFs in Figure 2A, with values downscaled by multiplication with the effect factor. A visualization of endpoint CFs for CO_2_ can be found in the Supporting Information, Figure S9. Similarly, the distributions for CO and CH_4_ are the same, with downscaled values as a result of the varying dissolution factors. All endpoint CFs can be found in Tables S9 and S10 in the Supporting Information. The globally aggregated endpoint CF for CO_2_ is 4.25 × 10^–14^ PDF yr/kg, for CO it is 3.70 × 10^–14^ PDF yr/kg, and for CH_4_ it is 3.54 × 10^–14^ PDF yr/kg_._

The range of the endpoint CFs presented here is significant enough to support the necessity of a spatially explicit midpoint model and an effect model with wide biological representation. The range of the CFs presented highlights the spatial variability in the sensitivity of marine ecosystems to acidification, reflecting differences in ocean chemistry, ecological composition, and species-level responses. Incorporating a wide biological representation ensures that the diverse vulnerabilities of species are captured, enabling more comprehensive and ecologically meaningful assessments. The CFs calculated in this study can be applied to any life cycle inventory containing CO_2_, CO, or CH_4_ emissions to the air.

Comprehensive models such as the one presented here are critical for informing targeted mitigation strategies and preserving ecosystem services vital to global biodiversity and human well-being. LCIA frameworks today are lacking in representation for marine impact categories, with some of the most common frameworks (e.g., GLAM or LC-IMPACT) having only 1–2 marine impact categories. While underrepresented, anthropogenic emissions can have significant impacts on marine ecosystems, as demonstrated in this study. For example, the maritime shipping industry emitted roughly 820 million tons of CO_2_ in 2023.^44^ Using the CF we have developed in this study, as well as the endpoint CF for climate change provided by LC-IMPACT,^45^ this emission would result in 3.48 × 10^–3^ PDF·yr from ocean acidification and 1.44 × 10^–3^ PDF·yr from climate change. The unit PDF·yr (potentially disappeared fraction of species over time) reflects a proportional loss of biodiversity risk across an ecosystem and not a literal share of species that go extinct. A value of 3.48 × 10^–3^ PDF·yr indicates a sustained pressure equivalent to 0.35% of species being under threat of disappearance for one year. In this case, the results suggest that the marine biodiversity risk from ocean acidification is nearly 2.5 times greater than that from climate change, based on the same CO_2_ emissions, underscoring the urgency of including ocean acidification impacts in LCA.

Using the same maritime shipping industry data, we can demonstrate the importance of spatially differentiated CFs. The Laptev Sea is an Arctic marine ecoregion along the coast of Siberia with the most severe midpoint CF of all of the regions. When paired with the polar effect factor to give us an “arctic” endpoint CF, maritime shipping emissions would cause 1.96 × 10^–2^ PDF·yr from ocean acidification. The Rio de la Plata is a tropical estuarine marine ecoregion feeding into the Atlantic Ocean with the least severe midpoint CF of all regions. When paired with the tropical effect factor, marine shipping emissions would cause 4.05 × 10^–5^ PDF·yr.

This example illustrates a nearly 200-fold difference in biodiversity risk depending on where emissions are ultimately absorbed. While the absolute number of species differs between these regions, the PDF metric represents a relative fraction of potentially affected species within each region, not an absolute species count. As such, comparisons of PDF values across regions remain meaningful in expressing relative ecosystem vulnerability, even if the underlying species’ richness varies. This underscores the need for spatial differentiation in CFs: the same emissions can result in significantly different biodiversity risks depending on the local sensitivity.

The chemical mechanism of ocean acidification has been well understood for decades; however, research on how it affects marine biology and ecosystems has grown rapidly in recent years and continues to expand. Some generalizations are agreed upon, such as strongly calcifying species being more sensitive to ocean acidification, but overall generalizations are difficult to apply, and research suggests that complicated mechanisms, including natural pH fluctuation, life stage, or costressors, affect species sensitivity in ways we do not yet fully understand. This makes it especially important to control for the elements that are more well-understood, such as the spatial dynamics of ocean acidification. Our study presents a novel endpoint model that captures the spatial aspect, as well as an effect model that expands the range of taxonomic groups and traditional binary classification of noncalcifying and calcifying, and that can continue to be built upon as new research on species sensitivity to ocean acidification is published.

Anthropogenic pressures on the ocean are growing rapidly, and at the same time, policies such as the EU’s Blue Economy represent the increasingly widespread goal to preserve marine environments. These dual realities make the representation of marine models in LCIA increasingly important. Our model can be added to commonly used frameworks to ameliorate the under-representation of marine impacts in LCIA and provides an important new perspective to assess the impacts of anthropogenic greenhouse gas emissions.

All FFs, FSFs, midpoint CFs, and endpoint CFs are downloadable via the Supporting Information. The effect factors are available in Table 1.

## Supplementary Material



## Notes

R and Python packages; version information for Python packages; literature sources for SSD data derivation; species representation by calcification type, region, and trophic level; FF values [Pa*yr/kg] per marine ecoregion; FF values [Pa*yr/kg] per FAO major fishing area; FSF values [pH/Pa] per marine ecoregion; FSF values [pH/Pa] per FAO major fishing area; midpoint CF values [pH*yr/kg] per marine ecoregion; midpoint CF values [pH*yr/kg] per marine ecoregion; data variability within MERs vs FAOs of spCO_2_ and pH; regional- and calcification-level SSD curves; visualization of endpoint CFs; endpoint CF values [PDF*yr/kg] per marine ecoregion; and endpoint CF values [PDF*yr/kg] per FAO (PDF)
