# Supplementary material for: From Global Emissions to Local Impacts: Spatially Explicit Modeling of Ocean Acidification in Life Cycle Assessment
Source: Environ Sci Technol. 2025 Oct 16;59(42):22508–17. doi: 10.1021/acs.est.5c02069 (PMC12573785; doi:10.1021/acs.est.5c02069)
Supplement: Supplementary file 1 [file es5c02069_si_001.pdf]

From Global Emissions to Local Impacts: Spatially Explicit Modeling of Ocean Acidification in life cycle assessment

Sedona R Anderson*†, Konstantin Stadler†, Francesca Verones†

†Industrial Ecology Programme, Department of Energy and Process Engineering, NTNU, 7491 Trondheim, Norway

*Phone : +47 41 25 79 83 ; E-mail: Sedona.anderson@ntnu.no

SUPPLEMENTARY INFORMATION

Contents Summary:

Total Pages: 32

Sections: 2

Tables: 10

Figures: 9

Section 1: References for packages used in R

(1) Hijmans, R. terra: Spatial Data Analysis; R package version 1.8-55, 2025. https://rspatial.org/.
(2) Wickham, H. ggplot2: Elegant Graphics for Data Analysis; Springer-Verlag: New York, 2016.
(3) Wickham, H.; François, R.; Henry, L.; Müller, K.; Vaughan, D. dplyr: A Grammar of Data Manipulation; R package version 1.1.4, 2025. https://dplyr.tidyverse.org.
(4) Pebesma, E.; Bivand, R. Spatial Data Science: With Applications in R; Chapman and Hall/CRC: 2023. https://doi.org/10.1201/9780429459016.
(5) Schauberger, P.; Walker, A. openxlsx: Read, Write and Edit xlsx Files; R package version 4.2.8, 2025. https://github.com/ycphs/openxlsx; https://ycphs.github.io/openxlsx/index.html.
(6) Wickham, H.; Bryan, J. readxl: Read Excel Files; R package version 1.4.5, 2025. https://github.com/tidyverse/readxl; https://readxl.tidyverse.org.
(7) Pierce, D. ncdf4: Interface to Unidata netCDF (Version 4 or Earlier) Format Data Files; R package version 1.24, 2025. https://CRAN.R-project.org/package=ncdf4.

Table S1: Version information for packages used in Python

**Package**	**Version**
xarray	2024.9.0
gdal	3.9.3
netCDF4	1.7.2
rasterio	1.4.1
matplotlib	3.9.2
rioxarray	0.19.0
numpy	2.1.2

Section 2: References for studies used to calculate pH_{50} and pH_{10} for use in SSD curves

(1) Cripps, G.; Lindeque, P.; Flynn, K. J. Have We Been Underestimating the Effects of Ocean Acidification in Zooplankton? Global Change Biology **2014**, 20 (11), 3377–3385. https://doi.org/10.1111/gcb.12582.
(2) Gazeau, F.; Quiblier, C.; Jansen, J. M.; Gattuso, J.-P.; Middelburg, J. J.; Heip, C. H. R. Impact of Elevated CO2 on Shellfish Calcification. Geophysical Research Letters **2007**, 34 (7). https://doi.org/10.1029/2006GL028554.
(3) Tegomo, F. A.; Zhong, Z.; Njomoue, A. P.; Okon, S. U.; Ullah, S.; Gray, N. A.; Chen, K.; Sun, Y.; Xiao, J.; Wang, L.; Ye, Y.; Huang, H.; Shao, Q. Experimental Studies on the Impact of the Projected Ocean Acidification on Fish Survival, Health, Growth, and Meat Quality; Black Sea Bream (Acanthopagrus Schlegelii), Physiological and Histological Studies. Animals **2021**, 11 (11), 3119. https://doi.org/10.3390/ani11113119.
(4) Crim, R. N.; Sunday, J. M.; Harley, C. D. G. Elevated Seawater CO2 Concentrations Impair Larval Development and Reduce Larval Survival in Endangered Northern Abalone (Haliotis Kamtschatkana). Journal of Experimental Marine Biology and Ecology **2011**, 400 (1), 272–277. https://doi.org/10.1016/j.jembe.2011.02.002.
(5) Inoue, M.; Suwa, R.; Suzuki, A.; Sakai, K.; Kawahata, H. Effects of Seawater pH on Growth and Skeletal U/Ca Ratios of Acropora Digitifera Coral Polyps. Geophysical Research Letters **2011**, 38 (12). https://doi.org/10.1029/2011GL047786.

(6) Gonzalez-Bernat, M. J.; Lamare, M.; Barker, M. Effects of Reduced Seawater pH on Fertilisation, Embryogenesis and Larval Development in the Antarctic Seastar Odontaster Validus. Polar Biol **2013**, 36 (2), 235–247. https://doi.org/10.1007/s00300-012-1255-7.
(7) Kurihara, H.; Shimode, S.; Shirayama, Y. Effects of Raised CO2 Concentration on the Egg Production Rate and Early Development of Two Marine Copepods (Acartia Steueri and Acartia Erythraea). Marine Pollution Bulletin **2004**, 49 (9), 721–727. https://doi.org/10.1016/j.marpolbul.2004.05.005.
(8) Faria, A. M.; Filipe, S.; Lopes, A. F.; Oliveira, A. P.; Gonçalves, E. J.; Ribeiro, L. Effects of High pCO2 on Early Life Development of Pelagic Spawning Marine Fish. Mar. Freshwater Res. **2017**, 68 (11), 2106–2114. https://doi.org/10.1071/MF16385.
(9) Renegar, D. A.; Riegl, B. M. Effect of Nutrient Enrichment and Elevated CO2 Partial Pressure on Growth Rate of Atlantic Scleractinian Coral Acropora Cervicornis. Marine Ecology Progress Series **2005**, 293, 69–76. https://doi.org/10.3354/meps293069.
(10) Comeau, S.; Cornwall, C. E.; McCulloch, M. T. Decoupling between the Response of Coral Calcifying Fluid pH and Calcification to Ocean Acidification. Sci Rep **2017**, 7 (1), 7573. https://doi.org/10.1038/s41598-017-08003-z.
(11) Sganga, D. E.; Dahlke, F. T.; Sørensen, S. R.; Butts, I. A. E.; Tomkiewicz, J.; Mazurais, D.; Servili, A.; Bertolini, F.; Politis, S. N. CO2 Induced Seawater Acidification Impacts Survival and Development of European Eel Embryos. PLOS ONE **2022**, 17 (4), e0267228. https://doi.org/10.1371/journal.pone.0267228.
(12) Collard, M.; Eeckhaut, I.; Dehairs, F.; Dubois, P. Acid–Base Physiology Response to Ocean Acidification of Two Ecologically and Economically Important Holothuroids from Contrasting Habitats, Holothuria Scabra and Holothuria Parva. Environ Sci Pollut Res **2014**, 21 (23), 13602–13614. https://doi.org/10.1007/s11356-014-3259-z.
(13) Small, D.; Calosi, P.; White, D.; Spicer, J. I.; Widdicombe, S. Impact of Medium-Term Exposure to CO2 Enriched Seawater on the Physiological Functions of the Velvet Swimming Crab Necora Puber. Aquatic Biology **2010**, 10 (1), 11–21. https://doi.org/10.3354/ab00266.
(14) Appelhans, Y.; Thomsen, J.; Opitz, S.; Pansch, C.; Melzner, F.; Wahl, M. Juvenile Sea Stars Exposed to Acidification Decrease Feeding and Growth with No Acclimation Potential. Mar. Ecol. Prog. Ser. **2014**, 509, 227–239. https://doi.org/10.3354/meps10884.
(15) Kamya, P. Z.; Dworjanyn, S. A.; Hardy, N.; Mos, B.; Uthicke, S.; Byrne, M. Larvae of the Coral Eating Crown-of-Thorns Starfish, Acanthaster Planci in a Warmer-High CO2 Ocean. Global Change Biology **2014**, 20 (11), 3365–3376. https://doi.org/10.1111/gcb.12530.
(16) Zlatkin, R. L.; Heuer, R. M. Ocean Acidification Affects Acid–Base Physiology and Behaviour in a Model Invertebrate, the California Sea Hare (Aplysia Californica). Royal Society Open Science **2019**, 6 (10), 191041. https://doi.org/10.1098/rsos.191041.
(17) Munday, P. L.; Dixson, D. L.; Donelson, J. M.; Jones, G. P.; Pratchett, M. S.; Devitsina, G. V.; Døving, K. B. Ocean Acidification Impairs Olfactory Discrimination and Homing Ability of a Marine Fish. Proceedings of the National Academy of Sciences **2009**, 106 (6), 1848–1852. https://doi.org/10.1073/pnas.0809996106.
(18) Stumpp, M.; Trübenbach, K.; Brennecke, D.; Hu, M. Y.; Melzner, F. Resource Allocation and Extracellular Acid–Base Status in the Sea Urchin Strongylocentrotus Droebachiensis in Response to CO2 Induced Seawater Acidification. Aquatic Toxicology **2012**, 110–111, 194–207. https://doi.org/10.1016/j.aquatox.2011.12.020.
(19) Comeau, S.; Jeffree, R.; Teyssié, J.-L.; Gattuso, J.-P. Response of the Arctic Pteropod Limacina Helicina to Projected Future Environmental Conditions. PLOS ONE **2010**, 5 (6), e11362. https://doi.org/10.1371/journal.pone.0011362.

(20) Frommel, A. Y.; Maneja, R.; Lowe, D.; Malzahn, A. M.; Geffen, A. J.; Folkvord, A.; Piatkowski, U.; Reusch, T. B. H.; Clemmesen, C. Severe Tissue Damage in Atlantic Cod Larvae under Increasing Ocean Acidification. Nature Clim Change **2012**, 2 (1), 42–46. https://doi.org/10.1038/nclimate1324.
(21) Okazaki, R. R.; Towle, E. K.; van Hooidonk, R.; Mor, C.; Winter, R. N.; Piggot, A. M.; Cunning, R.; Baker, A. C.; Klaus, J. S.; Swart, P. K.; Langdon, C. Species-Specific Responses to Climate Change and Community Composition Determine Future Calcification Rates of Florida Keys Reefs. Global Change Biology **2017**, 23 (3), 1023–1035. https://doi.org/10.1111/gcb.13481.
(22) Wisshak, M.; Schönberg, C. H. L.; Form, A.; Freiwald, A. Sponge Bioerosion Accelerated by Ocean Acidification across Species and Latitudes? Helgol Mar Res **2014**, 68 (2), 253–262. https://doi.org/10.1007/s10152-014-0385-4.
(23) Talmage, S. C.; Gobler, C. J. The Effects of Elevated Carbon Dioxide Concentrations on the Metamorphosis, Size, and Survival of Larval Hard Clams (Mercenaria Mercenaria), Bay Scallops (Argopecten Irradians), and Eastern Oysters (Crassostrea Virginica). Limnology and Oceanography **2009**, 54 (6), 2072–2080. https://doi.org/10.4319/lo.2009.54.6.2072.

Table S2. Total species representation in each calcification, region and trophic level category

**Category**	**Classification**	**Total**
Calcification	Non	10
	Slightly	6
	Strongly	16
Region	Polar	7
	Temperate	19
	Sub-tropical	15
	Tropical	13
Trophic Level	Primary consumer	20
	Secondary consumer	7
	Tertiary consumer	5

*Table S3: Fate factor values for CO_2, CH_4, and CO [Pa*yr/kg] per marine ecoregion*

**Marine ecoregion**	**CO_2**	**CH_4**	**CO**
Adriatic Sea	3.05E-12	2.55E-12	2.66E-12
Aegean Sea	3.05E-12	2.54E-12	2.65E-12
Agulhas Bank	2.87E-12	2.39E-12	2.50E-12
Alboran Sea	3.15E-12	2.63E-12	2.74E-12
Aleutian Islands	4.72E-12	3.94E-12	4.11E-12

Amazonia	3.69E-12	3.08E-12	3.21E-12
Amsterdam-St Paul	3.40E-12	2.84E-12	2.96E-12
Amundsen/Bellingshausen Sea	4.32E-12	3.61E-12	3.77E-12
Andaman and Nicobar Islands	3.19E-12	2.66E-12	2.77E-12
Andaman Sea Coral Coast	3.21E-12	2.68E-12	2.79E-12
Angolan	3.42E-12	2.85E-12	2.98E-12
Antarctic Peninsula	4.20E-12	3.50E-12	3.66E-12
Arabian (Persian) Gulf	3.57E-12	2.98E-12	3.11E-12
Arafura Sea	3.26E-12	2.72E-12	2.84E-12
Araucanian	3.86E-12	3.22E-12	3.36E-12
Arnhem Coast to Gulf of Carpenteria	3.30E-12	2.75E-12	2.87E-12
Auckland Island	4.01E-12	3.35E-12	3.49E-12
Azores Canaries Madeira	3.20E-12	2.67E-12	2.79E-12
Baffin Bay - Davis Strait	3.99E-12	3.32E-12	3.47E-12
Bahamian	2.86E-12	2.39E-12	2.49E-12
Baltic Sea	3.36E-12	2.80E-12	2.92E-12
Banda Sea	3.35E-12	2.79E-12	2.92E-12
Bassian	3.47E-12	2.89E-12	3.02E-12
Beaufort Sea - continental coast and shelf	5.48E-12	4.57E-12	4.77E-12
Beaufort-Amundsen-Viscount Melville-Queen Maud	4.96E-12	4.14E-12	4.32E-12
Bermuda	2.93E-12	2.45E-12	2.55E-12
Bight of Sofala/Swamp Coast	3.08E-12	2.57E-12	2.68E-12
Bismarck Sea	3.50E-12	2.92E-12	3.05E-12
Black Sea	2.50E-12	2.09E-12	2.18E-12
Bonaparte Coast	3.34E-12	2.79E-12	2.91E-12
Bounty and Antipodes Islands	3.89E-12	3.24E-12	3.39E-12
Bouvet Island	4.71E-12	3.93E-12	4.10E-12
Campbell Island	4.15E-12	3.46E-12	3.61E-12
Cape Howe	3.00E-12	2.50E-12	2.61E-12
Cape Verde	3.11E-12	2.59E-12	2.71E-12
Cargados Carajos/Tromelin Island	3.16E-12	2.64E-12	2.76E-12
Carolinian	2.87E-12	2.39E-12	2.50E-12
Celtic Seas	3.45E-12	2.88E-12	3.01E-12
Central and Southern Great Barrier Reef	2.89E-12	2.41E-12	2.52E-12
Central Chile	3.82E-12	3.18E-12	3.32E-12
Central Kuroshio Current	2.91E-12	2.43E-12	2.54E-12

Central New Zealand	3.22E-12	2.69E-12	2.81E-12
Central Peru	4.62E-12	3.85E-12	4.02E-12
Central Somali Coast	3.20E-12	2.67E-12	2.79E-12
Chagos	3.17E-12	2.64E-12	2.76E-12
Channels and Fjords of Southern Chile	4.42E-12	3.68E-12	3.85E-12
Chatham Island	3.30E-12	2.76E-12	2.88E-12
Chiapas-Nicaragua	3.45E-12	2.88E-12	3.01E-12
Chiloense	4.24E-12	3.53E-12	3.69E-12
Chukchi Sea	4.03E-12	3.36E-12	3.51E-12
Clipperton	3.30E-12	2.76E-12	2.88E-12
Cocos Islands	3.55E-12	2.96E-12	3.09E-12
Cocos-Keeling/Christmas Island	3.15E-12	2.63E-12	2.75E-12
Coral Sea	2.93E-12	2.44E-12	2.55E-12
Cortezian	4.17E-12	3.48E-12	3.63E-12
Crozet Islands	4.32E-12	3.60E-12	3.76E-12
Delagoa	2.91E-12	2.43E-12	2.54E-12
East African Coral Coast	3.15E-12	2.62E-12	2.74E-12
East Antarctic Dronning Maud Land	4.67E-12	3.90E-12	4.07E-12
East Antarctic Enderby Land	4.85E-12	4.05E-12	4.23E-12
East Antarctic Wilkes Land	4.64E-12	3.87E-12	4.04E-12
East Caroline Islands	3.24E-12	2.70E-12	2.82E-12
East China Sea	3.05E-12	2.54E-12	2.65E-12
East Greenland Shelf	3.67E-12	3.06E-12	3.20E-12
East Siberian Sea	5.76E-12	4.80E-12	5.01E-12
Easter Island	3.24E-12	2.70E-12	2.82E-12
Eastern Bering Sea	4.31E-12	3.59E-12	3.75E-12
Eastern Brazil	2.96E-12	2.47E-12	2.58E-12
Eastern Caribbean	2.91E-12	2.42E-12	2.53E-12
Eastern Galapagos Islands	4.03E-12	3.36E-12	3.51E-12
Eastern India	3.07E-12	2.56E-12	2.67E-12
Eastern Philippines	3.15E-12	2.63E-12	2.74E-12
Exmouth to Broome	3.22E-12	2.68E-12	2.80E-12
Faroe Plateau	3.72E-12	3.11E-12	3.24E-12
Fernando de Naronha and Atoll das Rocas	3.40E-12	2.83E-12	2.96E-12
Fiji Islands	2.82E-12	2.35E-12	2.46E-12
Floridian	2.89E-12	2.41E-12	2.52E-12
Gilbert/Ellis Islands	3.36E-12	2.81E-12	2.93E-12
Great Australian Bight	3.24E-12	2.70E-12	2.82E-12
Greater Antilles	2.97E-12	2.48E-12	2.59E-12
Guayaquil	4.09E-12	3.41E-12	3.56E-12
Guianan	3.23E-12	2.69E-12	2.81E-12

Gulf of Aden	3.42E-12	2.85E-12	2.98E-12
Gulf of Alaska	4.26E-12	3.55E-12	3.71E-12
Gulf of Guinea Central	3.08E-12	2.57E-12	2.68E-12
Gulf of Guinea Islands	3.10E-12	2.58E-12	2.70E-12
Gulf of Guinea South	3.41E-12	2.85E-12	2.97E-12
Gulf of Guinea Upwelling	3.16E-12	2.64E-12	2.76E-12
Gulf of Guinea West	3.08E-12	2.57E-12	2.69E-12
Gulf of Maine/Bay of Fundy	4.11E-12	3.43E-12	3.58E-12
Gulf of Oman	3.41E-12	2.84E-12	2.97E-12
Gulf of Papua	3.36E-12	2.80E-12	2.93E-12
Gulf of St. Lawrence - Eastern Scotian Shelf	4.99E-12	4.16E-12	4.35E-12
Gulf of Thailand	3.36E-12	2.80E-12	2.92E-12
Gulf of Tonkin	3.27E-12	2.73E-12	2.85E-12
Halmahera	3.39E-12	2.83E-12	2.95E-12
Hawaii	3.07E-12	2.56E-12	2.67E-12
Heard and Macdonald Islands	4.57E-12	3.81E-12	3.98E-12
High Arctic Archipelago	4.72E-12	3.93E-12	4.11E-12
Houtman	2.93E-12	2.44E-12	2.55E-12
Hudson Complex	4.03E-12	3.36E-12	3.51E-12
Humboldtian	4.13E-12	3.44E-12	3.60E-12
Ionian Sea	3.18E-12	2.66E-12	2.77E-12
Juan Fernandez and Desventuradas	3.70E-12	3.08E-12	3.22E-12
Kamchatka Shelf and Coast	4.41E-12	3.68E-12	3.85E-12
Kara Sea	4.41E-12	3.68E-12	3.84E-12
Kerguelen Islands	4.43E-12	3.69E-12	3.86E-12
Kermadec Island	2.99E-12	2.49E-12	2.60E-12
Lancaster Sound	4.26E-12	3.55E-12	3.71E-12
Laptev Sea	5.72E-12	4.77E-12	4.98E-12
Leeuwin	3.13E-12	2.61E-12	2.73E-12
Lesser Sunda	3.39E-12	2.83E-12	2.95E-12
Levantine Sea	3.04E-12	2.54E-12	2.65E-12
Line Islands	3.62E-12	3.02E-12	3.16E-12
Lord Howe and Norfolk Islands	2.91E-12	2.43E-12	2.53E-12
Macquarie Island	4.33E-12	3.61E-12	3.77E-12
Magdalena Transition	3.87E-12	3.23E-12	3.37E-12
Malacca Strait	3.27E-12	2.73E-12	2.85E-12
Maldives	3.11E-12	2.59E-12	2.71E-12
Malvinas/Falklands	3.98E-12	3.32E-12	3.47E-12
Manning-Hawkesbury	2.87E-12	2.39E-12	2.50E-12
Mariana Islands	3.00E-12	2.50E-12	2.61E-12
Marquesas	3.23E-12	2.69E-12	2.81E-12

Marshall Islands	3.08E-12	2.57E-12	2.68E-12
Mascarene Islands	3.02E-12	2.52E-12	2.63E-12
Mexican Tropical Pacific	3.49E-12	2.91E-12	3.04E-12
Namaqua	2.94E-12	2.45E-12	2.56E-12
Namib	3.62E-12	3.02E-12	3.15E-12
Natal	2.90E-12	2.42E-12	2.53E-12
New Caledonia	2.91E-12	2.43E-12	2.53E-12
Nicoya	3.65E-12	3.05E-12	3.18E-12
Ningaloo	3.04E-12	2.53E-12	2.64E-12
North American Pacific Fijordland	4.21E-12	3.51E-12	3.67E-12
North and East Barents Sea	3.21E-12	2.67E-12	2.79E-12
North and East Iceland	3.72E-12	3.11E-12	3.24E-12
North Greenland	3.87E-12	3.23E-12	3.37E-12
North Patagonian Gulfs	3.58E-12	2.99E-12	3.12E-12
North Sea	3.64E-12	3.04E-12	3.17E-12
Northeast Sulawesi	3.18E-12	2.65E-12	2.77E-12
Northeastern Brazil	3.16E-12	2.64E-12	2.75E-12
Northeastern Honshu	3.18E-12	2.65E-12	2.77E-12
Northeastern New Zealand	3.05E-12	2.55E-12	2.66E-12
Northern and Central Red Sea	3.51E-12	2.93E-12	3.06E-12
Northern Bay of Bengal	3.22E-12	2.68E-12	2.80E-12
Northern California	3.94E-12	3.29E-12	3.43E-12
Northern Galapagos Islands	3.45E-12	2.87E-12	3.00E-12
Northern Grand Banks - Southern Labrador	3.93E-12	3.28E-12	3.42E-12
Northern Gulf of Mexico	2.97E-12	2.48E-12	2.59E-12
Northern Labrador	3.66E-12	3.06E-12	3.19E-12
Northern Monsoon Current Coast	3.15E-12	2.63E-12	2.75E-12
Northern Norway and Finnmark	3.46E-12	2.88E-12	3.01E-12
Ogasawara Islands	2.98E-12	2.49E-12	2.60E-12
Oregon, Washington, Vancouver Coast and Shelf	3.88E-12	3.24E-12	3.38E-12
Oyashio Current	4.24E-12	3.53E-12	3.69E-12
Palawan/North Borneo	3.27E-12	2.73E-12	2.85E-12
Panama Bight	3.33E-12	2.78E-12	2.90E-12
Papua	3.53E-12	2.94E-12	3.07E-12
Patagonian Shelf	3.79E-12	3.16E-12	3.30E-12
Peter the First Island	4.52E-12	3.77E-12	3.94E-12
Phoenix/Tokelau/Northern Cook Islands	3.18E-12	2.65E-12	2.77E-12
Prince Edward Islands	4.22E-12	3.52E-12	3.68E-12
Puget Trough/Georgia Basin	4.10E-12	3.42E-12	3.57E-12

Rapa-Pitcairn	2.97E-12	2.48E-12	2.59E-12
Revillagigedos	3.41E-12	2.85E-12	2.97E-12
Rio de la Plata	3.21E-12	2.68E-12	2.80E-12
Rio Grande	3.07E-12	2.56E-12	2.67E-12
Ross Sea	4.16E-12	3.47E-12	3.62E-12
Saharan Upwelling	3.16E-12	2.64E-12	2.75E-12
Sahelian Upwelling	3.35E-12	2.79E-12	2.91E-12
Samoa Islands	2.83E-12	2.36E-12	2.46E-12
Sao Pedro and Sao Paulo Islands	3.34E-12	2.78E-12	2.91E-12
Scotian Shelf	4.26E-12	3.55E-12	3.71E-12
Sea of Japan/East Sea	3.16E-12	2.64E-12	2.76E-12
Sea of Okhotsk	4.03E-12	3.36E-12	3.51E-12
Seychelles	3.20E-12	2.67E-12	2.79E-12
Shark Bay	2.93E-12	2.45E-12	2.55E-12
Snares Island	3.77E-12	3.14E-12	3.28E-12
Society Islands	2.82E-12	2.35E-12	2.46E-12
Solomon Archipelago	3.23E-12	2.69E-12	2.81E-12
Solomon Sea	3.09E-12	2.58E-12	2.69E-12
South and West Iceland	3.77E-12	3.15E-12	3.29E-12
South Australian Gulfs	3.38E-12	2.82E-12	2.95E-12
South China Sea Oceanic Islands	3.28E-12	2.73E-12	2.85E-12
South European Atlantic Shelf	3.27E-12	2.73E-12	2.85E-12
South Georgia	4.68E-12	3.91E-12	4.08E-12
South India and Sri Lanka	3.17E-12	2.64E-12	2.76E-12
South Kuroshio	2.99E-12	2.49E-12	2.61E-12
South New Zealand	3.63E-12	3.03E-12	3.16E-12
South Orkney Islands	4.65E-12	3.88E-12	4.05E-12
South Sandwich Islands	4.62E-12	3.85E-12	4.02E-12
South Shetland Islands	4.72E-12	3.94E-12	4.11E-12
Southeast Madagascar	2.94E-12	2.45E-12	2.56E-12
Southeast Papua New Guinea	3.01E-12	2.51E-12	2.62E-12
Southeastern Brazil	2.99E-12	2.50E-12	2.61E-12
Southern California Bight	3.86E-12	3.22E-12	3.36E-12
Southern Caribbean	3.12E-12	2.60E-12	2.71E-12
Southern China	3.15E-12	2.63E-12	2.74E-12
Southern Cook/Austral Islands	2.85E-12	2.37E-12	2.48E-12
Southern Grand Banks - South Newfoundland	4.32E-12	3.60E-12	3.76E-12
Southern Gulf of Mexico	2.92E-12	2.44E-12	2.54E-12
Southern Java	3.27E-12	2.73E-12	2.85E-12
Southern Norway	3.60E-12	3.00E-12	3.14E-12

Southern Red Sea	4.18E-12	3.48E-12	3.64E-12
Southern Vietnam	3.33E-12	2.78E-12	2.90E-12
Southwestern Caribbean	3.07E-12	2.56E-12	2.67E-12
St. Helena and Ascension Islands	3.18E-12	2.65E-12	2.77E-12
Sulawesi Sea/Makassar Strait	3.32E-12	2.77E-12	2.89E-12
Sunda Shelf/Java Sea	3.45E-12	2.88E-12	3.01E-12
Three Kings-North Cape	2.98E-12	2.48E-12	2.59E-12
Tonga Islands	2.78E-12	2.32E-12	2.42E-12
Torres Strait Northern Great Barrier Reef	3.06E-12	2.55E-12	2.66E-12
Trindade and Martin Vaz Islands	3.07E-12	2.56E-12	2.67E-12
Tristan Gough	3.61E-12	3.02E-12	3.15E-12
Tuamotus	2.88E-12	2.40E-12	2.51E-12
Tunisian Plateau/Gulf of Sidra	3.15E-12	2.63E-12	2.74E-12
Tweed-Moreton	2.85E-12	2.38E-12	2.48E-12
Uruguay-Buenos Aires Shelf	3.22E-12	2.68E-12	2.80E-12
Vanuatu	2.91E-12	2.43E-12	2.54E-12
Virginian	3.38E-12	2.82E-12	2.94E-12
Weddell Sea	3.55E-12	2.96E-12	3.09E-12
West Caroline Islands	3.28E-12	2.74E-12	2.86E-12
West Greenland Shelf	3.46E-12	2.89E-12	3.01E-12
Western and Northern Madagascar	3.07E-12	2.56E-12	2.67E-12
Western Arabian Sea	3.52E-12	2.94E-12	3.07E-12
Western Bassian	3.50E-12	2.92E-12	3.05E-12
Western Caribbean	3.00E-12	2.50E-12	2.61E-12
Western Galapagos Islands	4.28E-12	3.57E-12	3.73E-12
Western India	2.92E-12	2.43E-12	2.54E-12
Western Mediterranean	3.27E-12	2.72E-12	2.84E-12
Western Sumatra	3.28E-12	2.73E-12	2.85E-12
White Sea	3.59E-12	3.00E-12	3.13E-12
Yellow Sea	3.54E-12	2.96E-12	3.09E-12

*Table S4: Fate factor values for CO_2, CH_4, and CO [Pa*yr/kg] per FAO major fishing area*

FAO major fishing area	CO_2	CH_4	CO
18	4.41E-12	3.68E-12	3.84E-12
21	3.42E-12	2.85E-12	2.98E-12
27	3.52E-12	2.94E-12	3.07E-12
31	2.92E-12	2.43E-12	2.54E-12
34	3.10E-12	2.59E-12	2.70E-12
41	3.42E-12	2.86E-12	2.98E-12
47	3.47E-12	2.90E-12	3.03E-12

48	4.49E-12	3.74E-12	3.91E-12
51	3.17E-12	2.64E-12	2.76E-12
57	3.54E-12	2.96E-12	3.09E-12
58	4.70E-12	3.92E-12	4.09E-12
61	3.49E-12	2.91E-12	3.04E-12
67	4.16E-12	3.47E-12	3.62E-12
71	3.14E-12	2.62E-12	2.73E-12
77	3.38E-12	2.82E-12	2.94E-12
81	3.68E-12	3.07E-12	3.21E-12
87	3.84E-12	3.20E-12	3.34E-12
88	4.62E-12	3.85E-12	4.02E-12

Table S5: Fate sensitivity factor values [pH/Pa] per marine ecoregions

Marine ecoregion	FSF
Adriatic Sea	-1.15E-02
Aegean Sea	-1.51E-02
Agulhas Bank	-1.03E-02
Alboran Sea	-1.06E-02
Aleutian Islands	-9.08E-03
Amazonia	-9.12E-03
Amsterdam-St Paul	-8.27E-03
Amundsen/Bellingshausen Sea	-1.19E-02
Andaman and Nicobar Islands	-9.55E-03
Andaman Sea Coral Coast	-8.52E-03
Angolan	-7.40E-03
Antarctic Peninsula	-1.22E-02
Arabian (Persian) Gulf	-9.46E-03
Arafura Sea	-5.09E-03
Araucanian	-8.73E-03
Arnhem Coast to Gulf of Carpenteria	-6.58E-03
Auckland Island	-9.83E-03
Azores Canaries Madeira	-8.33E-03
Baffin Bay - Davis Strait	-1.15E-02
Bahamian	-1.30E-02
Baltic Sea	-1.52E-02
Banda Sea	-8.01E-03
Bassian	-9.67E-03
Beaufort Sea - continental coast and shelf	-6.72E-03
Beaufort-Amundsen-Viscount Melville-Queen Maud	-1.15E-02
Bermuda	-1.07E-02
Bight of Sofala/Swamp Coast	-5.13E-03
Bismarck Sea	-6.42E-03
Black Sea	-1.01E-02

Bonaparte Coast	-4.67E-03
Bounty and Antipodes Islands	-1.01E-02
Bouvet Island	-1.04E-02
Campbell Island	-1.02E-02
Cape Howe	-9.56E-03
Cape Verde	-8.97E-03
Cargados Carajos/Tromelin Island	-9.30E-03
Carolinian	-1.22E-02
Celtic Seas	-9.39E-03
Central and Southern Great Barrier Reef	-7.27E-03
Central Chile	-7.53E-03
Central Kuroshio Current	-1.18E-02
Central New Zealand	-9.85E-03
Central Peru	-6.99E-03
Central Somali Coast	-9.34E-03
Chagos	-6.52E-03
Channels and Fjords of Southern Chile	-8.64E-03
Chatham Island	-9.94E-03
Chiapas-Nicaragua	-7.25E-03
Chiloense	-8.91E-03
Chukchi Sea	-1.15E-02
Clipperton	-8.11E-03
Cocos Islands	-6.79E-03
Cocos-Keeling/Christmas Island	-7.61E-03
Coral Sea	-1.03E-02
Cortezian	-7.72E-03
Crozet Islands	-9.01E-03
Delagoa	-7.61E-03
East African Coral Coast	-5.66E-03
East Antarctic Dronning Maud Land	-1.06E-02
East Antarctic Enderby Land	-1.17E-02
East Antarctic Wilkes Land	-1.08E-02
East Caroline Islands	-8.03E-03
East China Sea	-8.72E-03
East Greenland Shelf	-9.89E-03
East Siberian Sea	-1.21E-02
Easter Island	-1.03E-02
Eastern Bering Sea	-9.12E-03
Eastern Brazil	-8.22E-03
Eastern Caribbean	-1.27E-02
Eastern Galapagos Islands	-6.29E-03
Eastern India	-1.20E-02
Eastern Philippines	-7.11E-03

Exmouth to Broome	-6.21E-03
Faroe Plateau	-8.30E-03
Fernando de Naronha and Atoll das Rocas	-8.48E-03
Fiji Islands	-1.25E-02
Floridian	-1.26E-02
Gilbert/Ellis Islands	-7.53E-03
Great Australian Bight	-8.76E-03
Greater Antilles	-1.08E-02
Guayaquil	-7.43E-03
Guianan	-5.56E-03
Gulf of Aden	-8.85E-03
Gulf of Alaska	-9.27E-03
Gulf of Guinea Central	-7.18E-03
Gulf of Guinea Islands	-7.14E-03
Gulf of Guinea South	-6.83E-03
Gulf of Guinea Upwelling	-8.61E-03
Gulf of Guinea West	-7.76E-03
Gulf of Maine/Bay of Fundy	-1.31E-02
Gulf of Oman	-8.23E-03
Gulf of Papua	-5.46E-03
Gulf of St. Lawrence - Eastern Scotian Shelf	-1.14E-02
Gulf of Thailand	-4.71E-03
Gulf of Tonkin	-1.19E-02
Halmahera	-7.45E-03
Hawaii	-1.06E-02
Heard and Macdonald Islands	-1.15E-02
High Arctic Archipelago	-1.12E-02
Houtman	-9.78E-03
Hudson Complex	-7.15E-03
Humboldtian	-7.17E-03
Ionian Sea	-9.66E-03
Juan Fernandez and Desventuradas	-7.99E-03
Kamchatka Shelf and Coast	-9.42E-03
Kara Sea	-9.80E-03
Kerguelen Islands	-1.03E-02
Kermadec Island	-7.90E-03
Lancaster Sound	-1.28E-02
Laptev Sea	-1.60E-02
Leeuwin	-1.01E-02
Lesser Sunda	-8.02E-03
Levantine Sea	-9.29E-03
Line Islands	-7.49E-03
Lord Howe and Norfolk Islands	-8.20E-03
Macquarie Island	-7.79E-03

Magdalena Transition	-8.49E-03
Malacca Strait	-5.91E-03
Maldives	-9.06E-03
Malvinas/Falklands	-9.80E-03
Manning-Hawkesbury	-9.30E-03
Mariana Islands	-1.04E-02
Marquesas	-8.93E-03
Marshall Islands	-8.79E-03
Mascarene Islands	-8.96E-03
Mexican Tropical Pacific	-7.93E-03
Namaqua	-9.79E-03
Namib	-8.14E-03
Natal	-9.65E-03
New Caledonia	-8.62E-03
Nicoya	-6.69E-03
Ningaloo	-1.05E-02
North American Pacific Fijordland	-8.79E-03
North and East Barents Sea	-1.03E-02
North and East Iceland	-9.68E-03
North Greenland	-1.20E-02
North Patagonian Gulfs	-9.67E-03
North Sea	-1.43E-02
Northeast Sulawesi	-8.00E-03
Northeastern Brazil	-1.05E-02
Northeastern Honshu	-1.13E-02
Northeastern New Zealand	-9.40E-03
Northern and Central Red Sea	-9.20E-03
Northern Bay of Bengal	-1.26E-02
Northern California	-8.27E-03
Northern Galapagos Islands	-7.65E-03
Northern Grand Banks - Southern Labrador	-9.60E-03
Northern Gulf of Mexico	-1.17E-02
Northern Labrador	-1.28E-02
Northern Monsoon Current Coast	-8.71E-03
Northern Norway and Finnmark	-1.07E-02
Ogasawara Islands	-1.07E-02
Oregon, Washington, Vancouver Coast and Shelf	-8.09E-03
Oyashio Current	-1.14E-02
Palawan/North Borneo	-6.42E-03
Panama Bight	-6.38E-03
Papua	-6.70E-03
Patagonian Shelf	-9.55E-03
Peter the First Island	-9.42E-03

Phoenix/Tokelau/Northern Cook Islands	-8.33E-03
Prince Edward Islands	-1.11E-02
Puget Trough/Georgia Basin	-1.24E-02
Rapa-Pitcairn	-1.01E-02
Revillagigedos	-7.63E-03
Rio de la Plata	-1.47E-04
Rio Grande	-9.02E-03
Ross Sea	-1.25E-02
Saharan Upwelling	-9.89E-03
Sahelian Upwelling	-8.73E-03
Samoa Islands	-9.52E-03
Sao Pedro and Sao Paulo Islands	-7.10E-03
Scotian Shelf	-1.27E-02
Sea of Japan/East Sea	-1.32E-02
Sea of Okhotsk	-1.13E-02
Seychelles	-8.25E-03
Shark Bay	-7.55E-03
Snares Island	-9.96E-03
Society Islands	-7.36E-03
Solomon Archipelago	-7.30E-03
Solomon Sea	-7.77E-03
South and West Iceland	-9.24E-03
South Australian Gulfs	-8.49E-03
South China Sea Oceanic Islands	-1.07E-02
South European Atlantic Shelf	-8.69E-03
South Georgia	-9.92E-03
South India and Sri Lanka	-9.88E-03
South Kuroshio	-1.11E-02
South New Zealand	-9.83E-03
South Orkney Islands	-1.10E-02
South Sandwich Islands	-1.06E-02
South Shetland Islands	-1.13E-02
Southeast Madagascar	-9.31E-03
Southeast Papua New Guinea	-5.64E-03
Southeastern Brazil	-7.88E-03
Southern California Bight	-8.68E-03
Southern Caribbean	-8.25E-03
Southern China	-1.28E-02
Southern Cook/Austral Islands	-8.57E-03
Southern Grand Banks - South Newfoundland	-1.25E-02
Southern Gulf of Mexico	-1.06E-02
Southern Java	-8.15E-03
Southern Norway	-1.01E-02

Southern Red Sea	-7.17E-03
Southern Vietnam	-9.15E-03
Southwestern Caribbean	-9.15E-03
St. Helena and Ascension Islands	-1.09E-02
Sulawesi Sea/Makassar Strait	-6.60E-03
Sunda Shelf/Java Sea	-7.98E-03
Three Kings-North Cape	-9.25E-03
Tonga Islands	-8.94E-03
Torres Strait Northern Great Barrier Reef	-8.47E-03
Trindade and Martin Vaz Islands	-9.76E-03
Tristan Gough	-8.07E-03
Tuamotus	-8.89E-03
Tunisian Plateau/Gulf of Sidra	-1.10E-02
Tweed-Moreton	-9.68E-03
Uruguay-Buenos Aires Shelf	-9.83E-03
Vanuatu	-1.00E-02
Virginian	-1.28E-02
Weddell Sea	-1.27E-02
West Caroline Islands	-8.19E-03
West Greenland Shelf	-9.69E-03
Western and Northern Madagascar	-7.73E-03
Western Arabian Sea	-7.93E-03
Western Bassian	-8.93E-03
Western Caribbean	-6.73E-03
Western Galapagos Islands	-6.63E-03
Western India	-1.01E-02
Western Mediterranean	-7.79E-03
Western Sumatra	-7.15E-03
White Sea	-7.08E-03
Yellow Sea	-1.28E-02
Adriatic Sea	-1.15E-02

Table S6: Fate sensitivity factor values [pH/Pa] per FAO major fishing areas

FAO major fishing area	CO_2
18	-1.08E-02
21	-4.45E-03
27	-7.68E-03
31	-8.93E-03
34	-7.00E-03
41	-8.88E-03
47	-8.77E-03
48	-5.96E-03

51	-1.10E-02
57	-9.57E-03
58	-7.69E-03
61	-1.01E-02
67	-5.94E-03
71	-7.15E-03
77	-7.05E-03
81	-1.06E-02
87	-7.65E-03
88	-4.58E-03

*Table S7: Midpoint characterization factor values for CO_2, CH_4, and CO [pH*yr/kg] per marine ecoregion*

**Marine ecoregion**	**CO_2**	**CH_4**	**CO**
Adriatic Sea	3.51E-14	2.92E-14	3.05E-14
Aegean Sea	4.61E-14	3.84E-14	4.01E-14
Agulhas Bank	2.94E-14	2.46E-14	2.56E-14
Alboran Sea	3.33E-14	2.78E-14	2.90E-14
Aleutian Islands	4.29E-14	3.57E-14	3.73E-14
Amazonia	3.36E-14	2.81E-14	2.93E-14
Amsterdam-St Paul	2.81E-14	2.35E-14	2.45E-14
Amundsen/Bellingshausen Sea	5.13E-14	4.28E-14	4.47E-14
Andaman and Nicobar Islands	3.04E-14	2.54E-14	2.65E-14
Andaman Sea Coral Coast	2.74E-14	2.28E-14	2.38E-14
Angolan	2.53E-14	2.11E-14	2.20E-14
Antarctic Peninsula	5.13E-14	4.28E-14	4.46E-14
Arabian (Persian) Gulf	3.38E-14	2.82E-14	2.94E-14
Arafura Sea	1.66E-14	1.39E-14	1.45E-14
Araucanian	3.37E-14	2.81E-14	2.94E-14
Arnhem Coast to Gulf of Carpenteria	2.17E-14	1.81E-14	1.89E-14
Auckland Island	3.95E-14	3.29E-14	3.44E-14
Azores Canaries Madeira	2.66E-14	2.22E-14	2.32E-14
Baffin Bay - Davis Strait	4.59E-14	3.83E-14	4.00E-14
Bahamian	3.72E-14	3.10E-14	3.24E-14
Baltic Sea	5.10E-14	4.25E-14	4.44E-14
Banda Sea	2.68E-14	2.24E-14	2.34E-14
Bassian	3.35E-14	2.80E-14	2.92E-14
Beaufort Sea - continental coast and shelf	3.68E-14	3.07E-14	3.20E-14
Beaufort-Amundsen-Viscount Melville-Queen Maud	5.73E-14	4.78E-14	4.99E-14
Bermuda	3.14E-14	2.62E-14	2.73E-14
Bight of Sofala/Swamp Coast	1.58E-14	1.32E-14	1.38E-14
Bismarck Sea	2.25E-14	1.87E-14	1.96E-14
Black Sea	2.52E-14	2.10E-14	2.19E-14

Bonaparte Coast	1.56E-14	1.30E-14	1.36E-14
Bounty and Antipodes Islands	3.91E-14	3.26E-14	3.40E-14
Bouvet Island	4.90E-14	4.08E-14	4.27E-14
Campbell Island	4.22E-14	3.52E-14	3.67E-14
Cape Howe	2.86E-14	2.39E-14	2.49E-14
Cape Verde	2.79E-14	2.33E-14	2.43E-14
Cargados Carajos/Tromelin Island	2.94E-14	2.45E-14	2.56E-14
Carolinian	3.49E-14	2.91E-14	3.04E-14
Celtic Seas	3.24E-14	2.70E-14	2.82E-14
Central and Southern Great Barrier Reef	2.10E-14	1.75E-14	1.83E-14
Central Chile	2.88E-14	2.40E-14	2.50E-14
Central Kuroshio Current	3.44E-14	2.87E-14	3.00E-14
Central New Zealand	3.18E-14	2.65E-14	2.77E-14
Central Peru	3.23E-14	2.69E-14	2.81E-14
Central Somali Coast	2.99E-14	2.49E-14	2.60E-14
Chagos	2.07E-14	1.72E-14	1.80E-14
Channels and Fjords of Southern Chile	3.82E-14	3.18E-14	3.32E-14
Chatham Island	3.28E-14	2.74E-14	2.86E-14
Chiapas-Nicaragua	2.50E-14	2.09E-14	2.18E-14
Chiloense	3.77E-14	3.15E-14	3.29E-14
Chukchi Sea	4.62E-14	3.85E-14	4.02E-14
Clipperton	2.68E-14	2.23E-14	2.33E-14
Cocos Islands	2.41E-14	2.01E-14	2.10E-14
Cocos-Keeling/Christmas Island	2.40E-14	2.00E-14	2.09E-14
Coral Sea	3.01E-14	2.51E-14	2.62E-14
Cortezian	3.22E-14	2.68E-14	2.80E-14
Crozet Islands	3.89E-14	3.25E-14	3.39E-14
Delagoa	2.22E-14	1.85E-14	1.93E-14
East African Coral Coast	1.78E-14	1.48E-14	1.55E-14
East Antarctic Dronning Maud Land	4.97E-14	4.15E-14	4.33E-14
East Antarctic Enderby Land	5.66E-14	4.72E-14	4.93E-14
East Antarctic Wilkes Land	5.02E-14	4.19E-14	4.37E-14
East Caroline Islands	2.60E-14	2.17E-14	2.27E-14
East China Sea	2.66E-14	2.22E-14	2.31E-14
East Greenland Shelf	3.63E-14	3.03E-14	3.16E-14
East Siberian Sea	6.94E-14	5.79E-14	6.04E-14
Easter Island	3.32E-14	2.77E-14	2.89E-14
Eastern Bering Sea	3.93E-14	3.28E-14	3.42E-14
Eastern Brazil	2.44E-14	2.03E-14	2.12E-14
Eastern Caribbean	3.70E-14	3.09E-14	3.23E-14
Eastern Galapagos Islands	2.53E-14	2.11E-14	2.21E-14
Eastern India	3.68E-14	3.07E-14	3.20E-14
Eastern Philippines	2.24E-14	1.87E-14	1.95E-14

Exmouth to Broome	2.00E-14	1.67E-14	1.74E-14
Faroe Plateau	3.09E-14	2.58E-14	2.69E-14
Fernando de Naronha and Atoll das Rocas	2.88E-14	2.40E-14	2.51E-14
Fiji Islands	3.52E-14	2.94E-14	3.07E-14
Floridian	3.65E-14	3.04E-14	3.18E-14
Gilbert/Ellis Islands	2.53E-14	2.11E-14	2.21E-14
Great Australian Bight	2.83E-14	2.36E-14	2.47E-14
Greater Antilles	3.22E-14	2.68E-14	2.80E-14
Guayaquil	3.04E-14	2.53E-14	2.65E-14
Guianan	1.80E-14	1.50E-14	1.56E-14
Gulf of Aden	3.03E-14	2.52E-14	2.63E-14
Gulf of Alaska	3.94E-14	3.29E-14	3.44E-14
Gulf of Guinea Central	2.21E-14	1.84E-14	1.93E-14
Gulf of Guinea Islands	2.21E-14	1.84E-14	1.92E-14
Gulf of Guinea South	2.33E-14	1.94E-14	2.03E-14
Gulf of Guinea Upwelling	2.72E-14	2.27E-14	2.37E-14
Gulf of Guinea West	2.39E-14	2.00E-14	2.08E-14
Gulf of Maine/Bay of Fundy	5.40E-14	4.50E-14	4.70E-14
Gulf of Oman	2.80E-14	2.34E-14	2.44E-14
Gulf of Papua	1.83E-14	1.53E-14	1.60E-14
Gulf of St. Lawrence - Eastern Scotian Shelf	5.69E-14	4.75E-14	4.96E-14
Gulf of Thailand	1.58E-14	1.32E-14	1.38E-14
Gulf of Tonkin	3.88E-14	3.24E-14	3.38E-14
Halmahera	2.52E-14	2.10E-14	2.20E-14
Hawaii	3.24E-14	2.70E-14	2.82E-14
Heard and Macdonald Islands	5.24E-14	4.37E-14	4.56E-14
High Arctic Archipelago	5.29E-14	4.42E-14	4.61E-14
Houtman	2.87E-14	2.39E-14	2.50E-14
Hudson Complex	2.88E-14	2.41E-14	2.51E-14
Humboldtian	2.96E-14	2.47E-14	2.58E-14
Ionian Sea	3.08E-14	2.57E-14	2.68E-14
Juan Fernandez and Desventuradas	2.95E-14	2.46E-14	2.57E-14
Kamchatka Shelf and Coast	4.16E-14	3.47E-14	3.62E-14
Kara Sea	4.32E-14	3.60E-14	3.76E-14
Kerguelen Islands	4.56E-14	3.80E-14	3.97E-14
Kermadec Island	2.36E-14	1.97E-14	2.06E-14
Lancaster Sound	5.43E-14	4.53E-14	4.73E-14
Laptev Sea	9.16E-14	7.64E-14	7.98E-14
Leeuwin	3.17E-14	2.65E-14	2.77E-14
Lesser Sunda	2.72E-14	2.27E-14	2.37E-14
Levantine Sea	2.83E-14	2.36E-14	2.46E-14
Line Islands	2.71E-14	2.26E-14	2.36E-14
Lord Howe and Norfolk Islands	2.39E-14	1.99E-14	2.08E-14
Macquarie Island	3.37E-14	2.81E-14	2.94E-14

Magdalena Transition	3.28E-14	2.74E-14	2.86E-14
Malacca Strait	1.93E-14	1.61E-14	1.68E-14
Maldives	2.82E-14	2.35E-14	2.45E-14
Malvinas/Falklands	3.90E-14	3.25E-14	3.40E-14
Manning-Hawkesbury	2.67E-14	2.23E-14	2.33E-14
Mariana Islands	3.13E-14	2.61E-14	2.72E-14
Marquesas	2.88E-14	2.40E-14	2.51E-14
Marshall Islands	2.71E-14	2.26E-14	2.36E-14
Mascarene Islands	2.70E-14	2.25E-14	2.35E-14
Mexican Tropical Pacific	2.77E-14	2.31E-14	2.41E-14
Namaqua	2.88E-14	2.40E-14	2.50E-14
Namib	2.95E-14	2.46E-14	2.57E-14
Natal	2.80E-14	2.33E-14	2.44E-14
New Caledonia	2.51E-14	2.09E-14	2.18E-14
Nicoya	2.44E-14	2.04E-14	2.13E-14
Ningaloo	3.19E-14	2.66E-14	2.78E-14
North American Pacific Fijordland	3.70E-14	3.08E-14	3.22E-14
North and East Barents Sea	3.29E-14	2.74E-14	2.86E-14
North and East Iceland	3.60E-14	3.01E-14	3.14E-14
North Greenland	4.66E-14	3.88E-14	4.05E-14
North Patagonian Gulfs	3.46E-14	2.89E-14	3.02E-14
North Sea	5.21E-14	4.35E-14	4.54E-14
Northeast Sulawesi	2.54E-14	2.12E-14	2.22E-14
Northeastern Brazil	3.30E-14	2.75E-14	2.88E-14
Northeastern Honshu	3.58E-14	2.98E-14	3.12E-14
Northeastern New Zealand	2.87E-14	2.40E-14	2.50E-14
Northern and Central Red Sea	3.23E-14	2.69E-14	2.81E-14
Northern Bay of Bengal	4.04E-14	3.37E-14	3.52E-14
Northern California	3.26E-14	2.72E-14	2.84E-14
Northern Galapagos Islands	2.63E-14	2.20E-14	2.30E-14
Northern Grand Banks - Southern Labrador	3.77E-14	3.14E-14	3.28E-14
Northern Gulf of Mexico	3.49E-14	2.91E-14	3.04E-14
Northern Labrador	4.69E-14	3.91E-14	4.09E-14
Northern Monsoon Current Coast	2.75E-14	2.29E-14	2.39E-14
Northern Norway and Finnmark	3.69E-14	3.08E-14	3.22E-14
Ogasawara Islands	3.18E-14	2.65E-14	2.77E-14
Oregon, Washington, Vancouver Coast and Shelf	3.14E-14	2.62E-14	2.73E-14
Oyashio Current	4.82E-14	4.02E-14	4.19E-14
Palawan/North Borneo	2.10E-14	1.75E-14	1.83E-14
Panama Bight	2.12E-14	1.77E-14	1.85E-14
Papua	2.36E-14	1.97E-14	2.06E-14
Patagonian Shelf	3.62E-14	3.02E-14	3.15E-14
Peter the First Island	4.26E-14	3.55E-14	3.71E-14

Phoenix/Tokelau/Northern Cook Islands	2.65E-14	2.21E-14	2.30E-14
Prince Edward Islands	4.67E-14	3.89E-14	4.06E-14
Puget Trough/Georgia Basin	5.07E-14	4.23E-14	4.42E-14
Rapa-Pitcairn	3.00E-14	2.50E-14	2.61E-14
Revillagigedos	2.61E-14	2.17E-14	2.27E-14
Rio de la Plata	4.72E-16	3.94E-16	4.11E-16
Rio Grande	2.76E-14	2.31E-14	2.41E-14
Ross Sea	5.21E-14	4.35E-14	4.54E-14
Saharan Upwelling	3.13E-14	2.61E-14	2.72E-14
Sahelian Upwelling	2.92E-14	2.43E-14	2.54E-14
Samoa Islands	2.69E-14	2.24E-14	2.34E-14
Sao Pedro and Sao Paulo Islands	2.37E-14	1.98E-14	2.06E-14
Scotian Shelf	5.41E-14	4.51E-14	4.71E-14
Sea of Japan/East Sea	4.18E-14	3.49E-14	3.64E-14
Sea of Okhotsk	4.57E-14	3.81E-14	3.98E-14
Seychelles	2.64E-14	2.21E-14	2.30E-14
Shark Bay	2.21E-14	1.85E-14	1.93E-14
Snares Island	3.75E-14	3.13E-14	3.27E-14
Society Islands	2.08E-14	1.73E-14	1.81E-14
Solomon Archipelago	2.36E-14	1.96E-14	2.05E-14
Solomon Sea	2.40E-14	2.00E-14	2.09E-14
South and West Iceland	3.49E-14	2.91E-14	3.04E-14
South Australian Gulfs	2.87E-14	2.40E-14	2.50E-14
South China Sea Oceanic Islands	3.50E-14	2.92E-14	3.05E-14
South European Atlantic Shelf	2.84E-14	2.37E-14	2.47E-14
South Georgia	4.65E-14	3.88E-14	4.05E-14
South India and Sri Lanka	3.13E-14	2.61E-14	2.73E-14
South Kuroshio	3.32E-14	2.77E-14	2.89E-14
South New Zealand	3.57E-14	2.98E-14	3.11E-14
South Orkney Islands	5.12E-14	4.27E-14	4.46E-14
South Sandwich Islands	4.88E-14	4.07E-14	4.25E-14
South Shetland Islands	5.31E-14	4.43E-14	4.63E-14
Southeast Madagascar	2.74E-14	2.28E-14	2.38E-14
Southeast Papua New Guinea	1.70E-14	1.42E-14	1.48E-14
Southeastern Brazil	2.36E-14	1.97E-14	2.06E-14
Southern California Bight	3.35E-14	2.79E-14	2.92E-14
Southern Caribbean	2.57E-14	2.14E-14	2.24E-14
Southern China	4.04E-14	3.37E-14	3.52E-14
Southern Cook/Austral Islands	2.44E-14	2.03E-14	2.12E-14
Southern Grand Banks - South Newfoundland	5.42E-14	4.52E-14	4.72E-14
Southern Gulf of Mexico	3.10E-14	2.59E-14	2.70E-14
Southern Java	2.66E-14	2.22E-14	2.32E-14
Southern Norway	3.65E-14	3.05E-14	3.18E-14

Southern Red Sea	2.99E-14	2.50E-14	2.61E-14
Southern Vietnam	3.05E-14	2.55E-14	2.66E-14
Southwestern Caribbean	2.81E-14	2.34E-14	2.45E-14
St. Helena and Ascension Islands	3.48E-14	2.90E-14	3.03E-14
Sulawesi Sea/Makassar Strait	2.19E-14	1.83E-14	1.91E-14
Sunda Shelf/Java Sea	2.75E-14	2.30E-14	2.40E-14
Three Kings-North Cape	2.75E-14	2.30E-14	2.40E-14
Tonga Islands	2.48E-14	2.07E-14	2.16E-14
Torres Strait Northern Great Barrier Reef	2.59E-14	2.16E-14	2.25E-14
Trindade and Martin Vaz Islands	3.00E-14	2.50E-14	2.61E-14
Tristan Gough	2.92E-14	2.43E-14	2.54E-14
Tuamotus	2.56E-14	2.14E-14	2.23E-14
Tunisian Plateau/Gulf of Sidra	3.47E-14	2.90E-14	3.02E-14
Tweed-Moreton	2.76E-14	2.30E-14	2.40E-14
Uruguay-Buenos Aires Shelf	3.16E-14	2.64E-14	2.76E-14
Vanuatu	2.92E-14	2.44E-14	2.55E-14
Virginian	4.32E-14	3.60E-14	3.76E-14
Weddell Sea	4.51E-14	3.76E-14	3.93E-14
West Caroline Islands	2.69E-14	2.24E-14	2.34E-14
West Greenland Shelf	3.35E-14	2.80E-14	2.92E-14
Western and Northern Madagascar	2.37E-14	1.98E-14	2.06E-14
Western Arabian Sea	2.79E-14	2.33E-14	2.43E-14
Western Bassian	3.12E-14	2.61E-14	2.72E-14
Western Caribbean	2.02E-14	1.68E-14	1.76E-14
Western Galapagos Islands	2.84E-14	2.37E-14	2.47E-14
Western India	2.95E-14	2.46E-14	2.57E-14
Western Mediterranean	2.54E-14	2.12E-14	2.22E-14
Western Sumatra	2.34E-14	1.95E-14	2.04E-14
White Sea	2.55E-14	2.12E-14	2.22E-14
Yellow Sea	4.52E-14	3.77E-14	3.94E-14

*Table S8: Midpoint characterization factor values for CO_2, CH_4, and CO [pH*yr/kg] per FAO major fishing area*

**FAO major fishing area**	**CO_2**	**CH_4**	**CO**
18	4.78E-14	3.99E-14	4.16E-14
21	1.52E-14	1.27E-14	1.33E-14
27	2.70E-14	2.25E-14	2.35E-14
31	2.60E-14	2.17E-14	2.27E-14
34	2.17E-14	1.81E-14	1.89E-14
41	3.04E-14	2.53E-14	2.65E-14
47	3.05E-14	2.54E-14	2.66E-14
48	2.68E-14	2.23E-14	2.33E-14

51	3.48E-14	2.90E-14	3.03E-14
57	3.39E-14	2.83E-14	2.95E-14
58	3.61E-14	3.01E-14	3.15E-14
61	3.52E-14	2.94E-14	3.07E-14
67	2.47E-14	2.06E-14	2.15E-14
71	2.24E-14	1.87E-14	1.95E-14
77	2.38E-14	1.99E-14	2.07E-14
81	3.89E-14	3.25E-14	3.39E-14
87	2.94E-14	2.45E-14	2.56E-14
88	2.12E-14	1.76E-14	1.84E-14

Figure S1: Data variability within MERs vs FAOs of spCO2 and pH

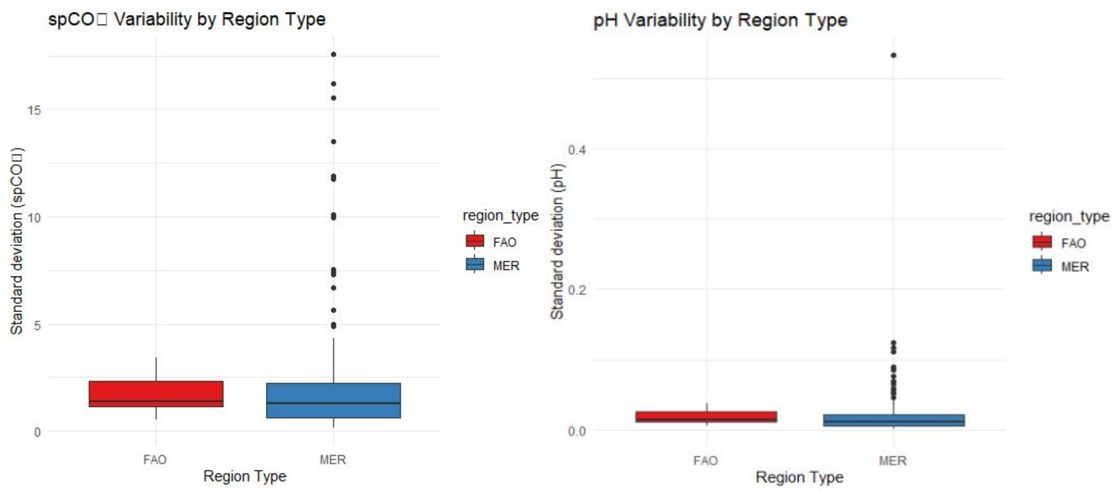

Though the FAO regions are larger than the MERs, with our data we are only looking at the top layer of ocean, which, for open ocean, is relatively homogenous. The coastal marine ecoregions, though often smaller, are more heterogenous than open ocean regions. Though there are data outliers, we chose to use averages rather than medians because the median would underrepresent the more common values within each marine ecoregion when such outliers are present. In this context, the average provides a more representative estimate of the general conditions and therefore a more appropriate basis for calculating characterization factors.

Figure S2: HC50 and HC20$_{pH10}$ SSD curves for strongly calcifying species

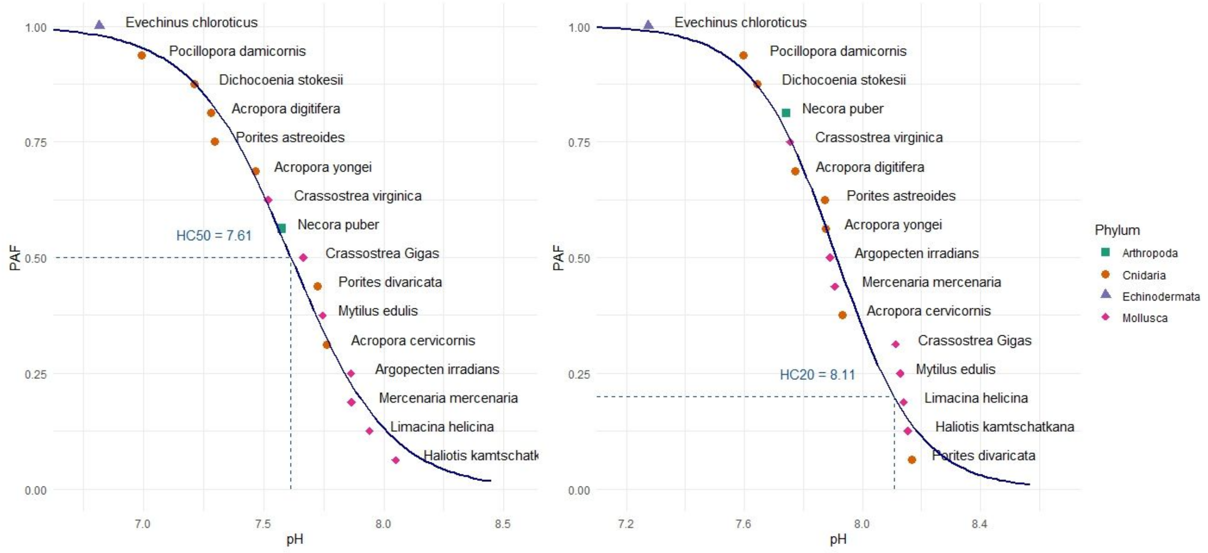

Figure S3: HC50 and HC20$_{pH10}$ SSD curves for slightly calcifying species

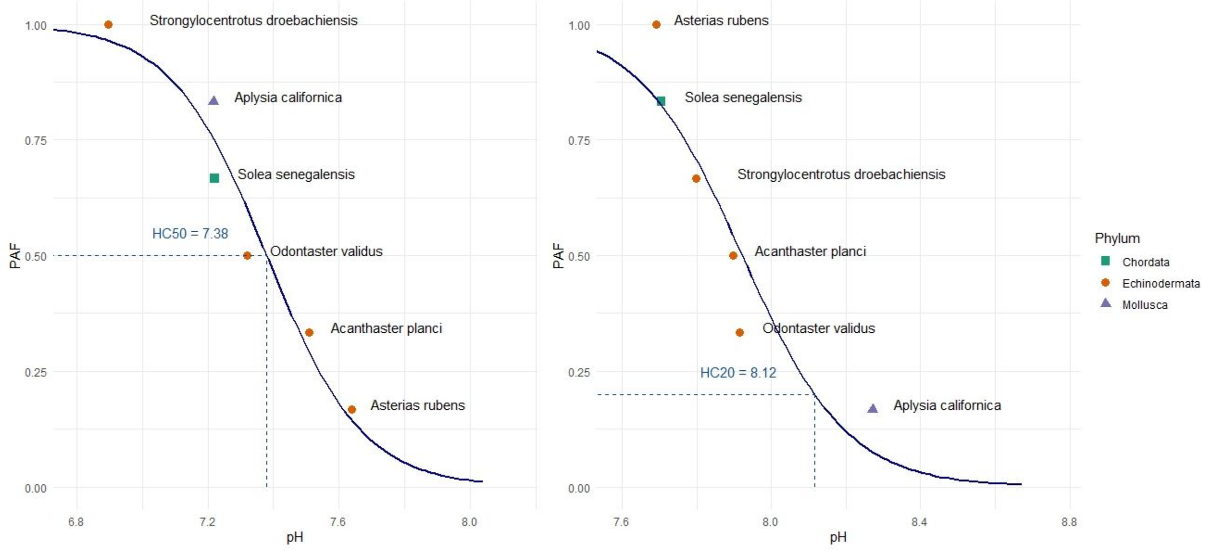

Figure S4: SSD curves for non-calcifying species

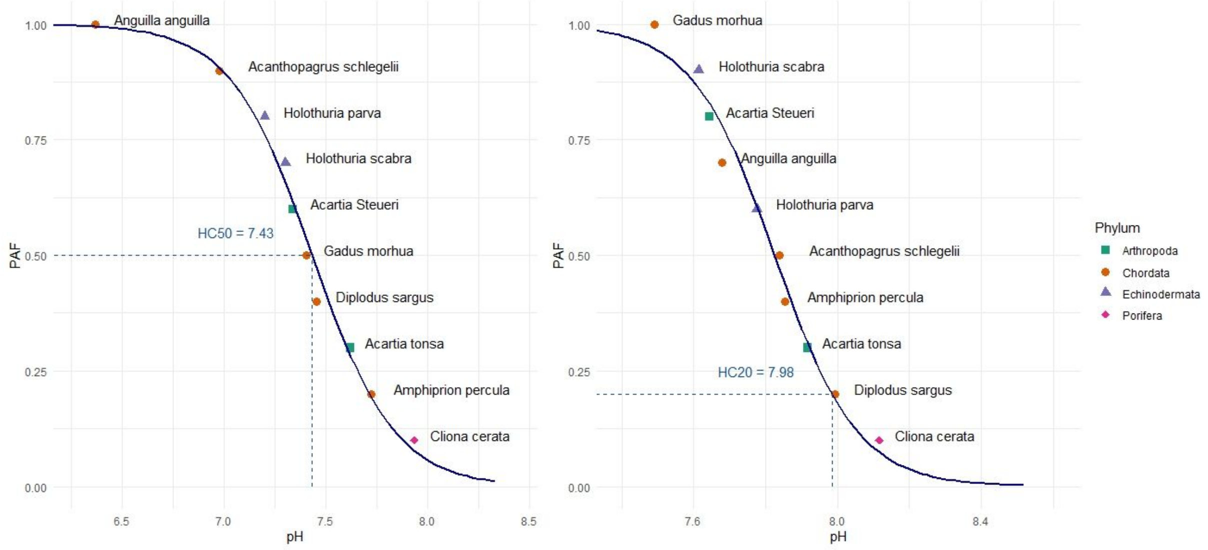

Figure S5: HC50 and HC20$_{pH10}$ SSD curves for tropical species

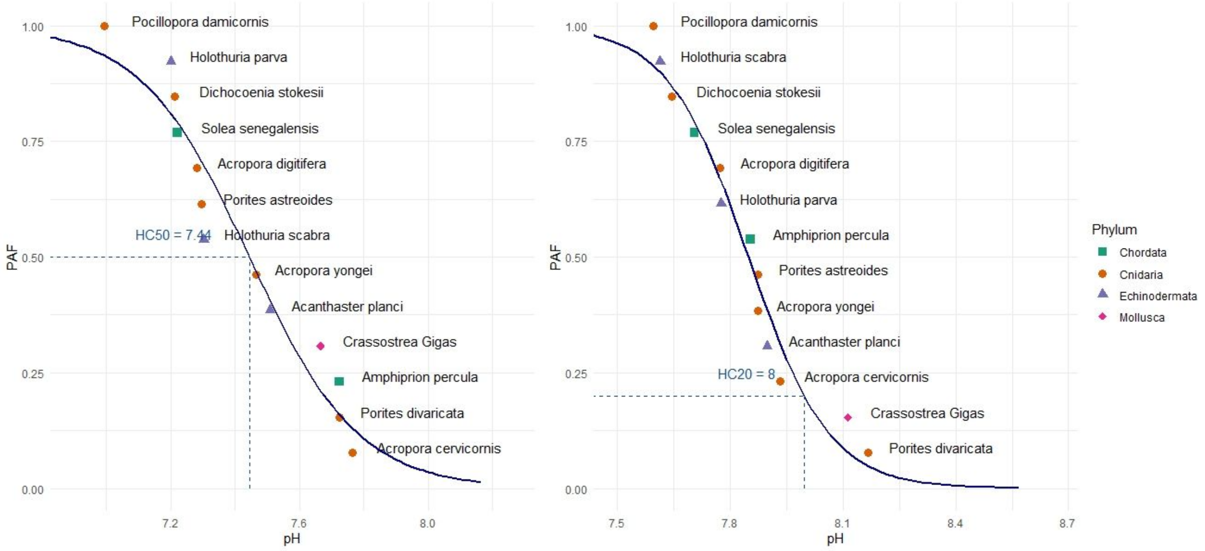

Figure S6: HC50 and HC20$_{pH10}$ SSD curves for subtropical species

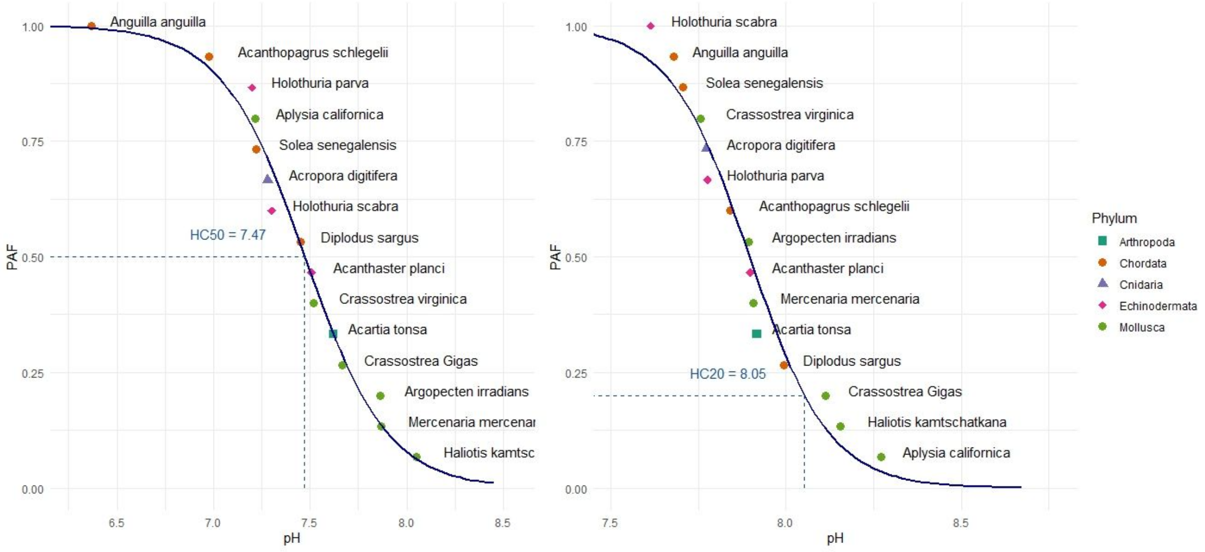

Figure S7: HC50 and HC20$_{pH10}$ SSD curves for temperate species

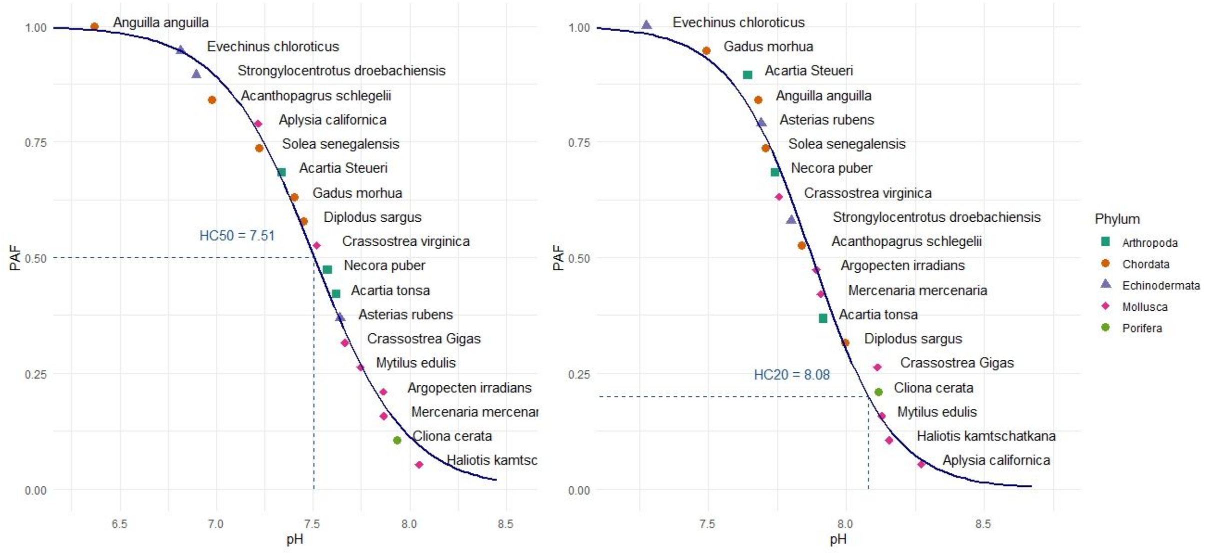

Figure S8: HC50 and $HC20_{pH10}$ SSD curves for polar species

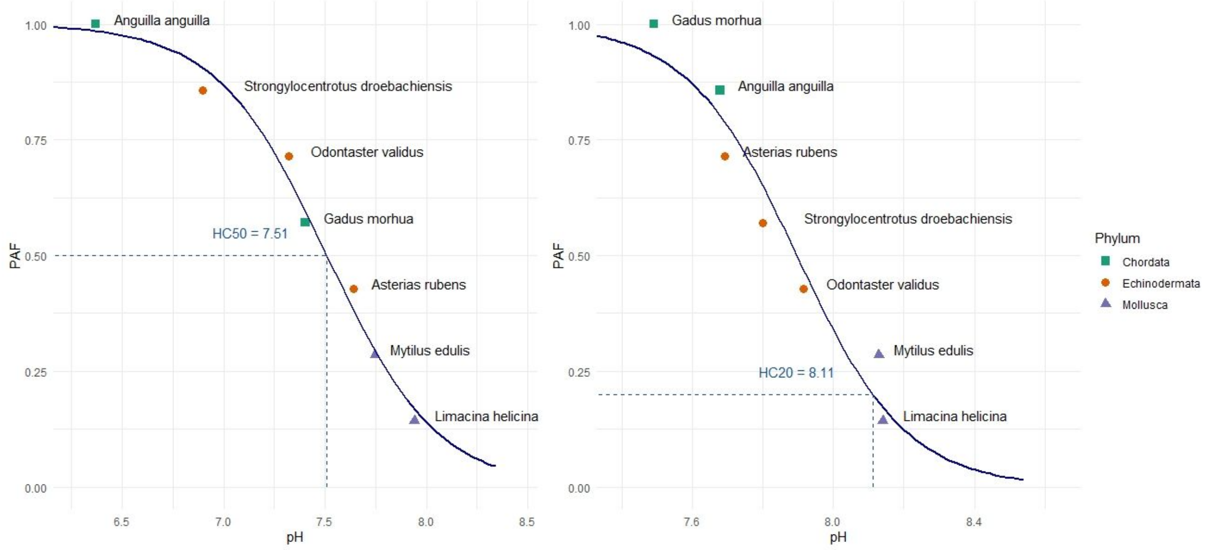

Figure S9: Visualization of endpoint characterization factors for CO_2. Change in PDF per kilogram CO_2 emitted [PDF yr/kg] (endpoint CF_{co2}) in FAO major fishing zones and marine ecoregions.

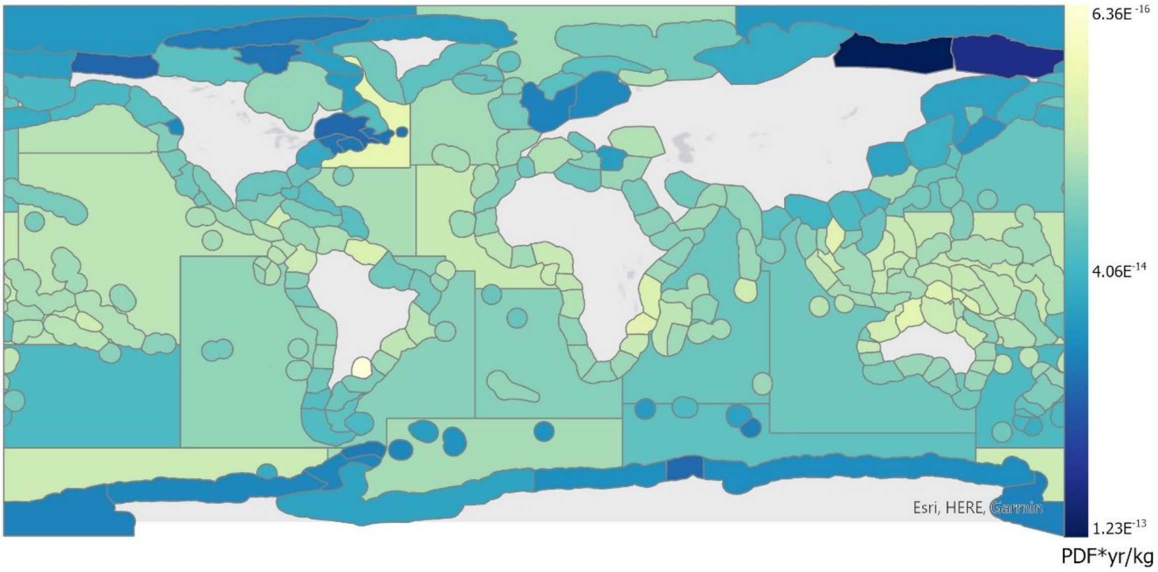

*Table S9: Endpoint characterization factor values for CO_2, CH_4, and CO [PDF*yr/kg] per marine ecoregion*

**Marine ecoregion**	**CO_2**	**CH_4**	**CO**
Adriatic Sea	4.72E-14	3.94E-14	4.11E-14
Aegean Sea	6.21E-14	5.18E-14	5.41E-14
Agulhas Bank	3.97E-14	3.31E-14	3.45E-14
Alboran Sea	4.49E-14	3.74E-14	3.91E-14
Aleutian Islands	5.77E-14	4.81E-14	5.03E-14
Amazonia	4.53E-14	3.78E-14	3.95E-14
Amsterdam-St Paul	3.79E-14	3.16E-14	3.30E-14
Amundsen/Bellingshausen Sea	6.91E-14	5.77E-14	6.02E-14
Andaman and Nicobar Islands	4.10E-14	3.42E-14	3.57E-14
Andaman Sea Coral Coast	3.68E-14	3.07E-14	3.21E-14
Angolan	3.41E-14	2.84E-14	2.97E-14
Antarctic Peninsula	6.90E-14	5.76E-14	6.01E-14
Arabian (Persian) Gulf	4.55E-14	3.80E-14	3.96E-14
Arafura Sea	2.24E-14	1.87E-14	1.95E-14
Araucanian	4.54E-14	3.79E-14	3.95E-14
Arnhem Coast to Gulf of Carpenteria	2.92E-14	2.44E-14	2.54E-14
Auckland Island	5.32E-14	4.43E-14	4.63E-14
Azores Canaries Madeira	3.59E-14	2.99E-14	3.13E-14
Baffin Bay - Davis Strait	6.18E-14	5.16E-14	5.38E-14
Bahamian	5.01E-14	4.18E-14	4.37E-14
Baltic Sea	6.87E-14	5.73E-14	5.98E-14
Banda Sea	3.61E-14	3.01E-14	3.15E-14
Bassian	4.51E-14	3.77E-14	3.93E-14

Beaufort Sea - continental coast and shelf	4.96E-14	4.13E-14	4.32E-14
Beaufort-Amundsen-Viscount Melville-Queen Maud	7.72E-14	6.44E-14	6.72E-14
Bermuda	4.23E-14	3.53E-14	3.68E-14
Bight of Sofala/Swamp Coast	2.13E-14	1.78E-14	1.86E-14
Bismarck Sea	3.03E-14	2.52E-14	2.63E-14
Black Sea	3.39E-14	2.83E-14	2.96E-14
Bonaparte Coast	2.10E-14	1.75E-14	1.83E-14
Bounty and Antipodes Islands	5.26E-14	4.39E-14	4.59E-14
Bouvet Island	6.60E-14	5.50E-14	5.75E-14
Campbell Island	5.68E-14	4.74E-14	4.95E-14
Cape Howe	3.86E-14	3.22E-14	3.36E-14
Cape Verde	3.76E-14	3.13E-14	3.27E-14
Cargados Carajos/Tromelin Island	3.96E-14	3.31E-14	3.45E-14
Carolinian	4.70E-14	3.92E-14	4.10E-14
Celtic Seas	4.37E-14	3.64E-14	3.80E-14
Central and Southern Great Barrier Reef	2.83E-14	2.36E-14	2.47E-14
Central Chile	3.87E-14	3.23E-14	3.37E-14
Central Kuroshio Current	4.64E-14	3.87E-14	4.04E-14
Central New Zealand	4.28E-14	3.57E-14	3.73E-14
Central Peru	4.35E-14	3.63E-14	3.79E-14
Central Somali Coast	4.03E-14	3.36E-14	3.51E-14
Chagos	2.78E-14	2.32E-14	2.43E-14
Channels and Fjords of Southern Chile	5.14E-14	4.29E-14	4.48E-14
Chatham Island	4.42E-14	3.69E-14	3.85E-14
Chiapas-Nicaragua	3.37E-14	2.81E-14	2.94E-14
Chiloense	5.08E-14	4.24E-14	4.43E-14
Chukchi Sea	6.22E-14	5.19E-14	5.42E-14
Clipperton	3.61E-14	3.01E-14	3.14E-14
Cocos Islands	3.25E-14	2.71E-14	2.83E-14
Cocos-Keeling/Christmas Island	3.23E-14	2.70E-14	2.82E-14
Coral Sea	4.05E-14	3.38E-14	3.53E-14
Cortezian	4.33E-14	3.62E-14	3.78E-14
Crozet Islands	5.24E-14	4.37E-14	4.57E-14
Delagoa	2.99E-14	2.49E-14	2.60E-14
East African Coral Coast	2.40E-14	2.00E-14	2.09E-14
East Antarctic Dronning Maud Land	6.70E-14	5.59E-14	5.83E-14
East Antarctic Enderby Land	7.62E-14	6.36E-14	6.64E-14
East Antarctic Wilkes Land	6.76E-14	5.64E-14	5.89E-14
East Caroline Islands	3.51E-14	2.92E-14	3.05E-14
East China Sea	3.58E-14	2.98E-14	3.12E-14
East Greenland Shelf	4.89E-14	4.08E-14	4.26E-14

East Siberian Sea	9.35E-14	7.80E-14	8.14E-14
Easter Island	4.48E-14	3.73E-14	3.90E-14
Eastern Bering Sea	5.29E-14	4.42E-14	4.61E-14
Eastern Brazil	3.28E-14	2.74E-14	2.86E-14
Eastern Caribbean	4.99E-14	4.16E-14	4.35E-14
Eastern Galapagos Islands	3.41E-14	2.85E-14	2.97E-14
Eastern India	4.96E-14	4.13E-14	4.32E-14
Eastern Philippines	3.01E-14	2.51E-14	2.63E-14
Exmouth to Broome	2.69E-14	2.25E-14	2.35E-14
Faroe Plateau	4.16E-14	3.47E-14	3.63E-14
Fernando de Naronha and Atoll das Rocas	3.88E-14	3.23E-14	3.38E-14
Fiji Islands	4.75E-14	3.96E-14	4.14E-14
Floridian	4.92E-14	4.10E-14	4.28E-14
Gilbert/Ellis Islands	3.41E-14	2.84E-14	2.97E-14
Great Australian Bight	3.82E-14	3.18E-14	3.33E-14
Greater Antilles	4.33E-14	3.61E-14	3.77E-14
Guayaquil	4.09E-14	3.41E-14	3.56E-14
Guianan	2.42E-14	2.02E-14	2.11E-14
Gulf of Aden	4.07E-14	3.40E-14	3.55E-14
Gulf of Alaska	5.31E-14	4.43E-14	4.63E-14
Gulf of Guinea Central	2.98E-14	2.48E-14	2.59E-14
Gulf of Guinea Islands	2.98E-14	2.48E-14	2.59E-14
Gulf of Guinea South	3.14E-14	2.62E-14	2.74E-14
Gulf of Guinea Upwelling	3.67E-14	3.06E-14	3.20E-14
Gulf of Guinea West	3.22E-14	2.69E-14	2.81E-14
Gulf of Maine/Bay of Fundy	7.27E-14	6.07E-14	6.33E-14
Gulf of Oman	3.78E-14	3.15E-14	3.29E-14
Gulf of Papua	2.47E-14	2.06E-14	2.15E-14
Gulf of St. Lawrence - Eastern Scotian Shelf	7.67E-14	6.39E-14	6.68E-14
Gulf of Thailand	2.13E-14	1.77E-14	1.85E-14
Gulf of Tonkin	5.23E-14	4.36E-14	4.56E-14
Halmahera	3.40E-14	2.84E-14	2.96E-14
Hawaii	4.36E-14	3.64E-14	3.80E-14
Heard and Macdonald Islands	7.05E-14	5.88E-14	6.14E-14
High Arctic Archipelago	7.13E-14	5.95E-14	6.21E-14
Houtman	3.86E-14	3.22E-14	3.36E-14
Hudson Complex	3.88E-14	3.24E-14	3.38E-14
Humboldtian	3.99E-14	3.33E-14	3.47E-14
Ionian Sea	4.14E-14	3.46E-14	3.61E-14
Juan Fernandez and Desventuradas	3.98E-14	3.32E-14	3.46E-14
Kamchatka Shelf and Coast	5.60E-14	4.67E-14	4.88E-14
Kara Sea	5.82E-14	4.85E-14	5.07E-14
Kerguelen Islands	6.14E-14	5.13E-14	5.35E-14
Kermadec Island	3.18E-14	2.65E-14	2.77E-14

Lancaster Sound	7.32E-14	6.10E-14	6.37E-14
Laptev Sea	1.23E-13	1.03E-13	1.07E-13
Leeuwin	4.28E-14	3.57E-14	3.73E-14
Lesser Sunda	3.66E-14	3.06E-14	3.19E-14
Levantine Sea	3.81E-14	3.18E-14	3.32E-14
Line Islands	3.66E-14	3.05E-14	3.18E-14
Lord Howe and Norfolk Islands	3.21E-14	2.68E-14	2.80E-14
Macquarie Island	4.54E-14	3.79E-14	3.95E-14
Magdalena Transition	4.42E-14	3.69E-14	3.85E-14
Malacca Strait	2.60E-14	2.17E-14	2.27E-14
Maldives	3.79E-14	3.16E-14	3.30E-14
Malvinas/Falklands	5.25E-14	4.38E-14	4.57E-14
Manning-Hawkesbury	3.60E-14	3.00E-14	3.13E-14
Mariana Islands	4.21E-14	3.51E-14	3.67E-14
Marquesas	3.88E-14	3.24E-14	3.38E-14
Marshall Islands	3.65E-14	3.04E-14	3.18E-14
Mascarene Islands	3.64E-14	3.04E-14	3.17E-14
Mexican Tropical Pacific	3.73E-14	3.11E-14	3.25E-14
Namaqua	3.87E-14	3.23E-14	3.37E-14
Namib	3.97E-14	3.31E-14	3.46E-14
Natal	3.77E-14	3.14E-14	3.28E-14
New Caledonia	3.38E-14	2.82E-14	2.94E-14
Nicoya	3.29E-14	2.74E-14	2.86E-14
Ningaloo	4.30E-14	3.59E-14	3.75E-14
North American Pacific Fijordland	4.98E-14	4.16E-14	4.34E-14
North and East Barents Sea	4.43E-14	3.69E-14	3.86E-14
North and East Iceland	4.85E-14	4.05E-14	4.23E-14
North Greenland	6.27E-14	5.23E-14	5.46E-14
North Patagonian Gulfs	4.66E-14	3.89E-14	4.06E-14
North Sea	7.02E-14	5.86E-14	6.12E-14
Northeast Sulawesi	3.43E-14	2.86E-14	2.98E-14
Northeastern Brazil	4.45E-14	3.71E-14	3.87E-14
Northeastern Honshu	4.82E-14	4.02E-14	4.20E-14
Northeastern New Zealand	3.87E-14	3.23E-14	3.37E-14
Northern and Central Red Sea	4.35E-14	3.63E-14	3.79E-14
Northern Bay of Bengal	5.44E-14	4.54E-14	4.74E-14
Northern California	4.39E-14	3.66E-14	3.82E-14
Northern Galapagos Islands	3.55E-14	2.96E-14	3.09E-14
Northern Grand Banks - Southern Labrador	5.08E-14	4.24E-14	4.42E-14
Northern Gulf of Mexico	4.70E-14	3.92E-14	4.09E-14
Northern Labrador	6.32E-14	5.27E-14	5.51E-14
Northern Monsoon Current Coast	3.70E-14	3.09E-14	3.22E-14
Northern Norway and Finnmark	4.97E-14	4.15E-14	4.33E-14
Ogasawara Islands	4.28E-14	3.57E-14	3.73E-14

Oregon, Washington, Vancouver Coast and Shelf	4.23E-14	3.53E-14	3.68E-14
Oyashio Current	6.49E-14	5.41E-14	5.65E-14
Palawan/North Borneo	2.83E-14	2.36E-14	2.46E-14
Panama Bight	2.86E-14	2.38E-14	2.49E-14
Papua	3.18E-14	2.65E-14	2.77E-14
Patagonian Shelf	4.87E-14	4.06E-14	4.24E-14
Peter the First Island	5.74E-14	4.79E-14	5.00E-14
Phoenix/Tokelau/Northern Cook Islands	3.56E-14	2.97E-14	3.10E-14
Prince Edward Islands	6.28E-14	5.24E-14	5.47E-14
Puget Trough/Georgia Basin	6.83E-14	5.70E-14	5.95E-14
Rapa-Pitcairn	4.04E-14	3.37E-14	3.51E-14
Revillagigedos	3.51E-14	2.93E-14	3.06E-14
Rio de la Plata	6.36E-16	5.30E-16	5.54E-16
Rio Grande	3.72E-14	3.11E-14	3.24E-14
Ross Sea	7.02E-14	5.86E-14	6.12E-14
Saharan Upwelling	4.21E-14	3.51E-14	3.67E-14
Sahelian Upwelling	3.93E-14	3.28E-14	3.42E-14
Samoa Islands	3.62E-14	3.02E-14	3.15E-14
Sao Pedro and Sao Paulo Islands	3.19E-14	2.66E-14	2.78E-14
Scotian Shelf	7.29E-14	6.08E-14	6.35E-14
Sea of Japan/East Sea	5.64E-14	4.70E-14	4.91E-14
Sea of Okhotsk	6.15E-14	5.13E-14	5.36E-14
Seychelles	3.56E-14	2.97E-14	3.10E-14
Shark Bay	2.98E-14	2.49E-14	2.60E-14
Snares Island	5.05E-14	4.22E-14	4.40E-14
Society Islands	2.80E-14	2.33E-14	2.44E-14
Solomon Archipelago	3.17E-14	2.65E-14	2.76E-14
Solomon Sea	3.23E-14	2.70E-14	2.82E-14
South and West Iceland	4.70E-14	3.92E-14	4.09E-14
South Australian Gulfs	3.87E-14	3.23E-14	3.37E-14
South China Sea Oceanic Islands	4.72E-14	3.94E-14	4.11E-14
South European Atlantic Shelf	3.83E-14	3.19E-14	3.33E-14
South Georgia	6.26E-14	5.22E-14	5.45E-14
South India and Sri Lanka	4.22E-14	3.52E-14	3.67E-14
South Kuroshio	4.48E-14	3.73E-14	3.90E-14
South New Zealand	4.80E-14	4.01E-14	4.18E-14
South Orkney Islands	6.89E-14	5.75E-14	6.00E-14
South Sandwich Islands	6.57E-14	5.48E-14	5.72E-14
South Shetland Islands	7.16E-14	5.97E-14	6.24E-14
Southeast Madagascar	3.69E-14	3.08E-14	3.21E-14
Southeast Papua New Guinea	2.29E-14	1.91E-14	1.99E-14
Southeastern Brazil	3.18E-14	2.65E-14	2.77E-14
Southern California Bight	4.51E-14	3.76E-14	3.93E-14

Southern Caribbean	3.46E-14	2.89E-14	3.02E-14
Southern China	5.44E-14	4.54E-14	4.74E-14
Southern Cook/Austral Islands	3.28E-14	2.74E-14	2.86E-14
Southern Grand Banks - South Newfoundland	7.30E-14	6.09E-14	6.36E-14
Southern Gulf of Mexico	4.18E-14	3.48E-14	3.64E-14
Southern Java	3.59E-14	2.99E-14	3.13E-14
Southern Norway	4.92E-14	4.11E-14	4.29E-14
Southern Red Sea	4.03E-14	3.36E-14	3.51E-14
Southern Vietnam	4.11E-14	3.43E-14	3.58E-14
Southwestern Caribbean	3.78E-14	3.16E-14	3.30E-14
St. Helena and Ascension Islands	4.68E-14	3.91E-14	4.08E-14
Sulawesi Sea/Makassar Strait	2.95E-14	2.46E-14	2.57E-14
Sunda Shelf/Java Sea	3.71E-14	3.09E-14	3.23E-14
Three Kings-North Cape	3.71E-14	3.09E-14	3.23E-14
Tonga Islands	3.35E-14	2.79E-14	2.91E-14
Torres Strait Northern Great Barrier Reef	3.48E-14	2.91E-14	3.03E-14
Trindade and Martin Vaz Islands	4.04E-14	3.37E-14	3.51E-14
Tristan Gough	3.93E-14	3.28E-14	3.42E-14
Tuamotus	3.45E-14	2.88E-14	3.01E-14
Tunisian Plateau/Gulf of Sidra	4.68E-14	3.90E-14	4.07E-14
Tweed-Moreton	3.71E-14	3.10E-14	3.23E-14
Uruguay-Buenos Aires Shelf	4.26E-14	3.55E-14	3.71E-14
Vanuatu	3.94E-14	3.28E-14	3.43E-14
Virginian	5.82E-14	4.85E-14	5.07E-14
Weddell Sea	6.08E-14	5.07E-14	5.29E-14
West Caroline Islands	3.62E-14	3.02E-14	3.15E-14
West Greenland Shelf	4.52E-14	3.77E-14	3.93E-14
Western and Northern Madagascar	3.19E-14	2.66E-14	2.78E-14
Western Arabian Sea	3.76E-14	3.14E-14	3.28E-14
Western Bassian	4.21E-14	3.51E-14	3.67E-14
Western Caribbean	2.72E-14	2.27E-14	2.37E-14
Western Galapagos Islands	3.82E-14	3.19E-14	3.33E-14
Western India	3.97E-14	3.31E-14	3.46E-14
Western Mediterranean	3.43E-14	2.86E-14	2.98E-14
Western Sumatra	3.16E-14	2.63E-14	2.75E-14
White Sea	3.43E-14	2.86E-14	2.99E-14
Yellow Sea	6.09E-14	5.08E-14	5.30E-14

*Table S10: Endpoint characterization factor values for CO_2, CH_4, and CO [PDF*yr/kg] per FAO major fishing areas*

FAO major fishing area	CO_2	CH_4	CO
18	6.44E-14	5.37E-14	5.61E-14

21	2.05E-14	1.71E-14	1.79E-14
27	3.64E-14	3.04E-14	3.17E-14
31	3.51E-14	2.93E-14	3.06E-14
34	2.93E-14	2.44E-14	2.55E-14
41	4.09E-14	3.41E-14	3.56E-14
47	4.11E-14	3.43E-14	3.58E-14
48	3.60E-14	3.01E-14	3.14E-14
51	4.69E-14	3.91E-14	4.08E-14
57	4.57E-14	3.81E-14	3.98E-14
58	4.87E-14	4.06E-14	4.24E-14
61	4.74E-14	3.95E-14	4.13E-14
67	3.32E-14	2.77E-14	2.90E-14
71	3.02E-14	2.52E-14	2.63E-14
77	3.21E-14	2.68E-14	2.79E-14
81	5.24E-14	4.37E-14	4.57E-14
87	3.95E-14	3.30E-14	3.44E-14
88	2.85E-14	2.38E-14	2.48E-14
